# Merging scleractinian genera: the overwhelming genetic similarity between solitary *Desmophyllum* and colonial *Lophelia*

**DOI:** 10.1186/s12862-016-0654-8

**Published:** 2016-05-18

**Authors:** Anna Maria Addamo, Agostina Vertino, Jaroslaw Stolarski, Ricardo García-Jiménez, Marco Taviani, Annie Machordom

**Affiliations:** Departamento de Biodiversidad y Biología Evolutiva, Museo Nacional de Ciencias Naturales (MNCN-CSIC), José Gutiérrez Abascal 2, 28006 Madrid, Spain; Dipartimento di Scienze dell’Ambiente e del Territorio e di Scienze della Terra, Università di Milano Bicocca (UNIMIB), Piazza della Scienza 4, 20126 Milan, Italy; Department of Geology Renard Centre of Marine Geology, Universiteit Ghent, Krijgslaan 281, B-9000 Ghent, Belgium; Polskiej Akademii Nauk, Instytut Paleobiologii, Twarda 51/55, PL-00-818 Warsaw, Poland; Consiglio Nazionale delle Ricerche, Istituto di Scienze Marine (ISMAR), Via Gobetti 101, 40129 Bologna, Italy; Biology Department, Woods Hole Oceanographic Institution, 266 Woods Hole Road, Woods Hole, 02543 MA USA; Stazione Zoologica Anton Dohrn, Villa Comunale, 80121 Naples, Italy

**Keywords:** Mitochondrial genome, Microsatellites, Genetic divergence, Skeletal plasticity, *Desmophyllum dianthus*, *Lophelia pertusa*

## Abstract

**Background:**

In recent years, several types of molecular markers and new microscale skeletal characters have shown potential as powerful tools for phylogenetic reconstructions and higher-level taxonomy of scleractinian corals. Nonetheless, discrimination of closely related taxa is still highly controversial in scleractinian coral research. Here we used newly sequenced complete mitochondrial genomes and 30 microsatellites to define the genetic divergence between two closely related azooxanthellate taxa of the family Caryophylliidae: solitary *Desmophyllum dianthus* and colonial *Lophelia pertusa*.

**Results:**

In the mitochondrial control region, an astonishing 99.8 % of nucleotides between *L. pertusa* and *D. dianthus* were identical. Variability of the mitochondrial genomes of the two species is represented by only 12 non-synonymous out of 19 total nucleotide substitutions. Microsatellite sequence (37 loci) analysis of *L. pertusa* and *D. dianthus* showed genetic similarity is about 97 %. Our results also indicated that *L. pertusa* and *D. dianthus* show high skeletal plasticity in corallum shape and similarity in skeletal ontogeny, micromorphological (septal and wall granulations) and microstructural characters (arrangement of rapid accretion deposits, thickening deposits).

**Conclusions:**

Molecularly and morphologically, the solitary *Desmophyllum* and the dendroid *Lophelia* appear to be significantly more similar to each other than other unambiguous coral genera analysed to date. This consequently leads to ascribe both taxa under the generic name *Desmophyllum* (priority by date of publication). Findings of this study demonstrate that coloniality may not be a robust taxonomic character in scleractinian corals.

**Electronic supplementary material:**

The online version of this article (doi:10.1186/s12862-016-0654-8) contains supplementary material, which is available to authorized users.

## Background

Mitogenomics, or the analysis of complete mitochondrial genomic data sets, is a powerful tool used in a wide range of organisms to improve phylogenetic estimations, reconstruct robust phylogenies and resolve long-standing phylogenetic uncertainties [1–6]. Scleractinian mitochondrial genomes are estimated to be evolving 10–20 times slower than vertebrate ones, and five times slower than scleractinian nuclear genomes [7, 8], suggesting their limited application for species-level phylogenetics and population genetics [9, 10]. Nevertheless, they were reported as a useful tool for detecting population variability and structure [11, 12]. Furthermore, mitochondrial genome rearrangements occur relatively rarely and have been useful in resolving evolutionary relationships of closely related species, particularly in Scleractinia [13–19].

*Desmophyllum dianthus* (Esper, 1794) and *Lophelia pertusa* (Linneus, 1758) are azooxanthellate scleractinian corals living in cold waters worldwide, with the exception of the polar seas [20–22]. Both species belong to the polyphyletic family Caryophylliidae that is represented by several molecular clusters within Robusta [23, 24], one of three major molecular clades of scleractinian corals [25]. According to recent studies the family Caryophylliidae includes, besides *Desmophyllum* and *Lophelia*, numerous (ca. 70) modern species of nominal genera as *Caryophyllia*, *Crispatotrochus* Tenison-Woods, 1878, *Dasmosmilia* Pourtales, 1880, *Pourtalosmilia* Duncan, 1884, and *Stenocyathus* Pourtalès, 1868 (traditionally classified as representative of Guyniidae, see also [26]).

While *D. dianthus* is one out of three existing species (*D. quinarium* Tenison-Woods, 1879 and *D. striatum* Cairns, 1979) of the genus *Desmophyllum* Ehrenberg, 1834, *L. pertusa* is monotypic for the genus *Lophelia* Milne Edwards & Haime, 1849.

**Fig. 1:**
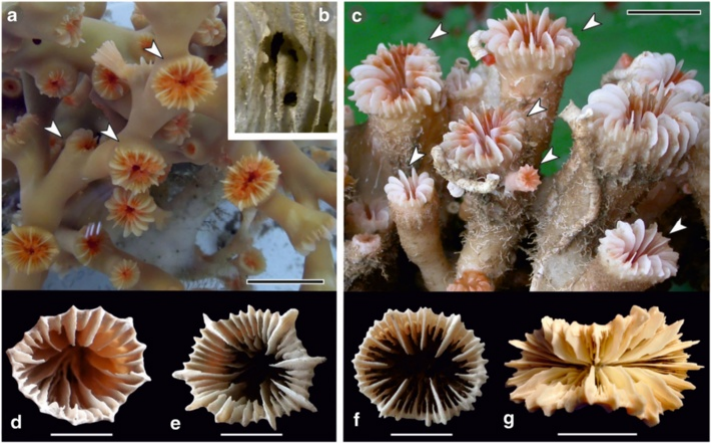
Colony and corallites of *Lophelia pertusa* and coralla of *Desmophyllum dianthus*. **a**. Branches of a live colony (multiple connected polyps) of *L. pertusa* (Moira Mounds, Porcupine Seabight, NE Atlantic; UniMiB-MM15). White arrows indicate examples of interconnected single polyps/corallites. **b**. Detail of the inner wall of a corallite of *L. pertusa*; note the holes that internally connect the soft tissues of two corallites of the same colony; these holes are missing in the wall of aggregated coralla of *D. dianthus*. **c**. Live aggregation of several solitary coralla of *D. dianthus* (Bari Canyon, Adriatic Sea, Mediterranean, dive 108, METEOR 70–1 cruise; sample held at Senckenberg am Meer, Germany). The skeletons of the coralla are secondarily fused, there is no connection between polyps (orange); each polyp/corallum (white arrows) results from a distinct larva settled on the parental skeleton. **d**-**e**. Calicular views of two corallites of *Lophelia*; at comparable sizes septa show different distribution pattern and size. **f**-**g**. Calicular views of two coralla of *Desmophyllum dianthus* showing high intraspecific morphological variability; **d**. UniMiB-SGC4, South Gulf of Cadiz; **e**. UniMiB-SML5, Ionian Sea, Mediterranean; **f**. UniMiB-SML8, Ionian Sea, Mediterranean; **g**. USNM 92612, Sagami Bay, Japan. Scale bars: **a**,**c**,**g**. 2 cm, **b**. 2 mm, **d**-**f**. 1 cm

*Desmophyllum dianthus* is a solitary but gregarious scleractinian species that actively contributes to the accretion of cold-water coral build-ups. Planulae of this species preferentially settle on parental skeleton, producing "branching" structures that, in some cases, can be mistaken as irregular colonies (Fig. 1a). The skeleton of this species is extremely variable, from very thin-walled to massive and from subcylindrical to trochoid and greatly flared (Fig. 1c, f, g, [20, 27–30]). *Lophelia pertusa* is a colonial species, forming bush-like colonies that can exceed one metre in diameter. It is the most common azooxanthellate frame-building scleractinian and the main component of the densest and most extensive cold-water coral bioconstructions known thus far (e.g. Norwegian continental shelf [22, 31]). Although less irregular than *D. dianthus,* the skeleton of *L. pertusa* is quite variable both in colonial pattern and calicular size and shape (Fig. 1a, b, d, e, [20, 27–29]). Both species typically occur in deep-water environments where they often share the same habitat, as documented in modern and Pleistocene settings in the Atlantic Ocean and Mediterranean Sea [e.g., 20, 28, 31–36]. However, one of the two species can dominate a distinct ecological niche within the same area (e.g. “solitary coral facies” [35, 36]) or in different areas of the same basin [37–39]. Occasionally, only one of the two species is present in some environments: e.g., *D. dianthus* forms the dense shallow-water bioconstructions in the Chilean fjords [40, 41], whereas *L. pertusa* dominates the Norwegian "Cold-Water Coral (CWC) reefs" [42, 43].

Though identified as two distinct genera, morphological and genetic similarities have been previously reported between *Desmophyllum* and *Lophelia* [20, 24, 44] and references therein [45], suggesting an ambiguous taxonomic status that requires confirmation. The main objective of this study is to establish a genetic fingerprint and clarify the phylogenetic relationship between *D. dianthus* and *L. pertusa*. New data consisting of complete mitochondrial genomes and other molecular markers, including previously analysed protein-coding and non-coding genes, were used to provide a robust molecular framework for reaching clear interpretations. We also provide further ontogenetic, microstructural, macro- and micromorphological evidence of the high degree of skeletal similarity between *L. pertusa* and *D. dianthus*.

## Results

### Genetic analysis

**Fig. 2:**
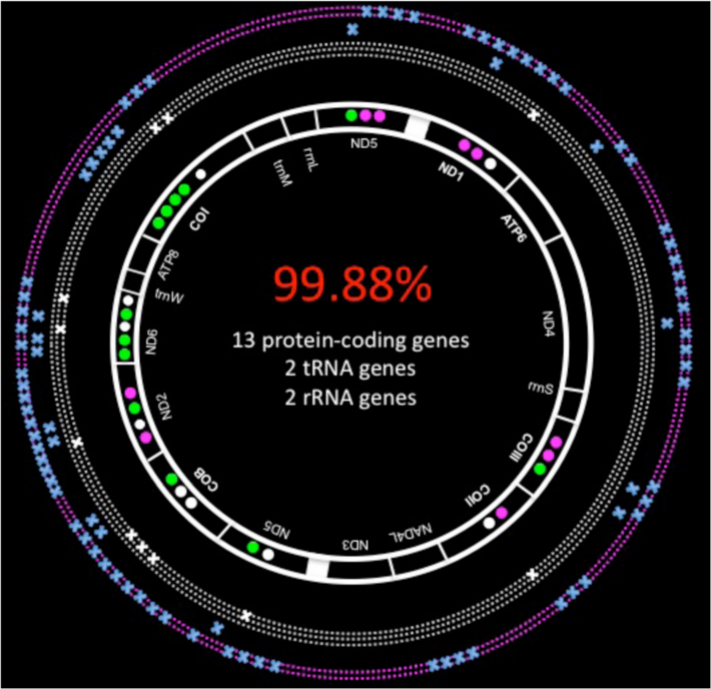
Mitochondrial sequences, gene arrangement and comparison between two *Desmophyllum dianthus* (from Mediterranean Sea and Chilean fjord, circle of purple dots) and three *Lophelia pertusa* (from Mediterranean Sea and Norwegian fjords, circle of white dots) specimens. The mitochondrial genomes *of D. dianthus* and *L. pertusa* show 99.88 % of genetic similarity. Nucleotide substitutions obtained in this study are indicated at the intra- and interspecific levels (blue cross). Intraspecific variability of the mitochondrial genome of *L. pertusa* (white cross) is attributed to the study of Flot et al. (2013) [50]. Non-synonymous substitutions found at interspecific (green point) and intraspecific levels (purple point for *D. dianthus*; white point for *L. pertusa*) are represented for each gene from which they were obtained

The mitochondrial genome of *D. dianthus*, with a length of between 16,229 and 16,310 base pairs (bp), had a nucleotide composition with a GC content of 35 %, similar to other corals [16, 46]. The mitochondrial genome rearrangement of *D. dianthus* was the same as described for *L. pertusa* [15]: the mitogenome contained 13 protein-coding genes, 2 transfer RNA genes, 2 ribosomal RNA genes, and a group I intron, which interrupted the *nd5* gene. This group consisted of one ribosomal (*rns*) and seven protein-coding genes (Fig. 2). Nearly all protein-coding genes had methionine (ATG) as the translation initiation codon (except *cob* and *nad2*, which had TAT and TTA, respectively), and TAA or TAG as the stop codon. The two largest non-coding regions were between the *nad5* and *cob* genes, consisting of the putative control region [15], and the *nad6* and *trnW* genes. The putative control region was responsible for the mitogenome length variation observed at both inter- and intraspecific levels: small insertions and deletions (INDELs) ranging from 72 bp to 150 bp in length were detected in *L. pertusa* (16,150 bp), and the Italian (16,229 bp) and Chilean (16,310 bp) specimens of *D. dianthus* (see Additional file 1.1).

Comparison of the two *D. dianthus* and three *L. pertusa* mitogenomes (excluding the putative control region) showed that genomic variability was represented by 86 nucleotide substitutions, of which 22 were non-synonymous (Table 1, see Additional file 1.2a and 1.2b). Intraspecific variability between *D. dianthus* individuals from the Mediterranean Sea and Chilean fjord was based on 9 non-synonymous substitutions of 67 mutations, with 99.58 % genetic similarity. Except for the INDELs in the putative control region, astonishing genetic similarity was found between *L. pertusa* and *D. dianthus*: only 12 substitutions out of 19 were observed over 13 coding genes; the remaining 99.88 % nucleotides were identical.

**Table 1:** Pairwise species non-synonymous substitutions with nucleotide (NT) and amino acid (AA) location

				*Dd432-LpKC875348*				*Dd432-LpKC875349*				*Dd432-LpFR821799*				*Dd432Dd636Dd432Dd636*				*Dd636LpKC875348Dd636LpKC875348*				*Dd636LpKC875348Dd636LpKC875348*				*Dd636LpFR821799Dd636LpFR821799*			
#	AA	NT	Gene	AA				AA				AA	AA				AA				AA				AA						
1	26	77	*nad5*	26	R	==>	K	26	R	==>	K	26	R	==>	K					26	R	==>	K	26	R	==>	K	26	R	==>	K
2	38	113	*nad5*	38	T	==>	I	38	T	==>	I	38	T	==>	I	38	T	==>	I												
3	165	493	*nad5*	165	V	==>	I	165	V	==>	I	165	V	==>	I	165	V	==>	I												
4	351	1051	*nad1*													351	K	==>	Q	351	Q	==>	K	351	Q	==>	K	351	Q	==>	K
5	427	1280	*nad1*	427	C	==>	F	427	C	==>	F					427	C	==>	F									427	F	==>	C
6	1403	4208	*cox3*													1403	N	==>	S	1403	S	==>	N	1403	S	==>	N	1403	S	==>	N
7	1408	4225	*cox3*	1408	G	==>	S	1408	G	==>	S	1408	G	==>	S	1409	G	==>	S												
8	1499	4498	*cox3*	1499	S	==>	G	1499	S	==>	G	1499	S	==>	G					1500	S	==>	G	1500	S	==>	G	1500	S	==>	G
9	1685	5056	*cox2*	1685	G	==>	R	1685	G	==>	R	1685	G	==>	R	1686	G	==>	R												
10	2289	6865	*nad5*									2288	E	==>	K													2289	E	==>	K
11	2471	7411	*cob*									2470	T	==>	P													2471	T	==>	P
12	2698	8097	*cob*	2698	L	==>	F	2698	L	==>	F									2699	L	==>	F	2699	L	==>	F				
13	2863	8589	*nad2*													2863	F	==>	L	2863	L	==>	F	2863	L	==>	F	2863	L	==>	F
14	2930	8789	*nad2*													2930	H	==>	R	2930	R	==>	H	2930	R	==>	H	2930	R	==>	H
15	3054	9161	*nad2*									3053	L	==>	S													3054	L	==>	S
16	3087	9260	*nad6*									3086	L	==>	P													3087	L	==>	P
17	3191	9574	*nad6*	3191	Y	==>	H													3192	Y	==>	H								
18	3221	9664	*nad6*	3221	L	==>	I	3221	L	==>	I	3221	L	==>	I					3222	L	==>	I	3222	L	==>	I	3222	L	==>	I
19	3348	10046	*cox1*	3348	A	==>	V	3348	A	==>	V	3348	A	==>	V					3349	A	==>	V	3349	A	==>	V	3349	A	==>	V
20	3354	10064	*cox1*	3354	V	==>	A	3354	V	==>	A	3354	V	==>	A					3355	V	==>	A	3355	V	==>	A	3355	V	==>	A
21	3389	10166	*cox1*									3388	S	==>	F													3389	S	==>	F
22	3713	11141	*cox1*	3713	R	==>	K	3713	R	==>	K	3713	R	==>	K					3714	R	==>	K	3714	R	==>	K				

*Dd* Desmoplyllum dianthus, *Lp* Lophelia pertusa

The dN/dS values obtained from pairwise comparisons between the mitochondrial protein-coding regions from individuals of both species ranged from 0.13 to 0.30. Higher substitution ratios, due to mathematical artefacts (e.g. when only one non-synonymous substitution occurs), were found between specimens of *L. pertusa* (Table 2).

**Table 2:** Computation of non-synonymous (dN) and synonymous (dS) substitutions between mitochondrial protein-coding genes of *D. dianthus* (*Dd*) and *L. pertusa* (*Lp*) using one approximate method (NG) and tree maximum-likelihood methods (GY-HKY; MS; MA) (Zhang et al. 2006) [79]

Pairwise Sequence	Method	Ka = dN	Ks = dS	Ka/Ks	*P*-value(Fisher)	Length	Substitutions	S-substitutions	*N*-substitutions
*Dd432-Dd636*	NG	0.00105426	0.00738853	0.14269	6.38E-02	11127	28	19	9
	GY-HKY	0.00103991	0.00780277	0.13328	3.46E-03	11127	28	19.0493	8.9507
	MS	0.00120913	0.00911783	0.13261	2.04E-02	11127	28	16.8877	11.1123
	MA	0.00111943	0.00849681	0.13175	2.57E-03	11127	28	18.0081	9.9919
*Dd432-LpKC875348*	NG	0.00151378	0.00618744	0.24465	0.000194732	11193	29	16	13
	GY-HKY	0.00147761	0.00678233	0.21786	1.27E-01	11193	29	16.0446	12.9554
	MS	0.00145719	0.00716942	0.20325	5.85E-01	11193	29	16.0453	12.9547
	MA	0.00149592	0.0071832	0.20825	2.11E + 00	11193	29	15.5980	13.4020
*Dd432-LpFR821799*	NG	0.00174798	0.00657818	0.26572	0.000305985	11187	32	17	15
	GY-HKY	0.00174679	0.00666052	0.26226	0.000133266	11187	32	17.0509	14.9491
	MS	0.0017217	0.00701057	0.24559	2.37E + 00	11187	32	17.0472	14.9528
	MA	0.00180584	0.00717326	0.25175	0.000138073	11187	32	15.9324	16.0676
*Dd432-LpKC875349*	NG	0.00139724	0.00618725	0.22583	0.000111185	11193	28	16	12
	GY-HKY	0.00135939	0.00686159	0.19812	5.55E-01	11193	28	16.0428	11.9572
	MS	0.00134202	0.00722641	0.18571	2.64E-01	11193	28	16.0436	11.9564
	MA	0.00138454	0.00726459	0.19059	9.52E-01	11193	28	15.5516	12.4484
*Dd636-LpFR821799 *	NG	0.00187087	0.00542254	0.34502	0.00136247	11154	30	14	16
	GY-HKY	0.00182133	0.00600644	0.30323	0.000711538	11154	30	14.0360	15.9640
	MS	0.0019595	0.00650537	0.30121	0.00171439	11154	30	11.8204	18.1796
	MA	0.00189377	0.00631826	0.29973	0.00217506	11154	30	12.8315	17.1685
*Dd636-LpKC875348*	NG	0.00140188	0.00542062	0.25862	0.00106907	11160	26	14	12
	GY-HKY	0.00134558	0.00632621	0.21270	2.50E + 00	11160	26	14.0360	11.9640
	MS	0.00134558	0.00632621	0.21270	2.50E + 00	11160	26	14.0360	11.9640
	MA	0.00137335	0.00649319	0.21151	6.87E + 00	11160	26	13.6246	12.3754
*Dd636-LpKC875349*	NG	0.00128497	0.00542045	0.23706	0.000405305	11160	25	14	11
	GY-HKY	0.00122824	0.0064301	0.19101	1.05E + 00	11160	25	14.0343	10.9657
	MS	0.00122824	0.0064301	0.19101	1.05E + 00	11160	25	14.0343	10.9657
	MA	0.00124788	0.00657364	0.18983	3.68E-01	11160	25	13.7456	11.2544
*LpKC875348-LpFR821799*	NG	0.00092881	0.000768297	1.20892	0.954969	11223	10	2	8
	GY-HKY	0.00088951	0.000903263	0.98477	0.929985	11223	10	1.9987	8.0013
	MS	0.00088951	0.000903263	0.98477	0.929985	11223	10	1.9987	8.0013
	MA	0.000874244	0.000974582	0.89705	0.605808	11223	10	1.9436	8.0564
*LpKC875348-LpKC875349*	NG	0.000115971	NA	NA	NA	11229	1	NA	1
	GY-HKY	0.000117817	2.36E-06	50.00000	0.367879	11229	1	0.0066	0.9934
	MS	0.000117817	2.36E-06	50.00000	0.367879	11229	1	0.0066	0.9934
	MA	0.000108301	2.17E-06	50.00000	0.367879	11229	1	0.0044	0.9956
*LpKC875349-LpFR821799*	NG	0.000812653	0.000768274	1.05776	0.941351	11223	9	2	7
	GY-HKY	0.000778073	0.000903374	0.86130	0.589175	11223	9	1.9299	7.0701
	MS	0.000778073	0.000903374	0.86130	0.589175	11223	9	1.9299	7.0701
	MA	0.000756522	0.00100953	0.74938	0.272516	11223	9	2.0420	6.9580

Based on the estimation of uncorrected *p*-distances among different scleractinian families and genera, genetic divergence ranged from 4 to 8 % between genera and 0.2 to 1 % between species (see Additional file 2). The genetic distance between *Lophelia* and *Desmophyllum* genera was estimated to be 0.8 %, the same value obtained when the two *D. dianthus* individuals were compared.

Estimations for the putative control region were performed separately. Within this region, pairwise comparisons between 9 *D. dianthus* and 5 *L. pertusa* individuals showed overlapping genetic distance ranges, from 9 to 14 % between genera and 0.3 to 14 % at the intraspecific level (Table 3).

**Table 3:** Genetic divergence between *D. dianthus* (*Dd*) and *L. pertusa* (*Lp*) individuals using only putative control region sequences

#	Individuals	1	2	3	4	5	6	7	8	9	10	11	12	13	14
1	*Lp*FR821799	–													
2	*Lp*KC875348	0.004	–												
3	*Lp*KC875349	0.004	0.000	–											
4	*Lp*IRL296	0.002	0.004	0.004	–										
5	*Lp*SML272	0.002	0.003	0.003	0.000	–									
6	*Dd*ARG472	0.108	0.110	0.110	0.107	0.090	–								
7	*Dd*AUS566	0.103	0.104	0.104	0.100	0.082	0.018	–							
8	*Dd*MNZ601	0.098	0.100	0.100	0.098	0.093	0.132	0.125	–						
9	*Dd*SML62	0.114	0.113	0.113	0.114	0.110	0.115	0.106	0.034	–					
10	*Dd*ADR635	0.101	0.102	0.102	0.102	0.101	0.127	0.123	0.031	0.009	–				
11	*Dd*ADR636	0.102	0.103	0.103	0.103	0.103	0.130	0.126	0.032	0.011	0.003	–			
12	*Dd*IJC433	0.088	0.090	0.090	0.088	0.087	0.104	0.100	0.086	0.103	0.088	0.090	–		
13	*Dd*ILC681	0.091	0.093	0.093	0.091	0.090	0.125	0.120	0.143	0.107	0.147	0.149	0.000	–	
14	*Dd*IJC432	0.096	0.098	0.098	0.098	0.090	0.132	0.128	0.147	0.104	0.144	0.146	0.000	0.002	–

Microsatellite sequence analysis showed that 30 microsatellite markers, developed for *D. dianthus* [47], successfully genotyped *L. pertusa* with clear peak profiles. In addition, *L. pertusa* individuals from the Mediterranean Sea and North Atlantic Ocean presented the same allele size range as *D. dianthus* (see Additional file 1.3, Additional file 3). Moreover, average microsatellite sequence identities between *L. pertusa* and *D. dianthus* were about 97 % similar, based on multiple BLAST alignments for 1368 separate pairwise comparisons*.*

### Skeletal analysis

The morphological analysis carried out in this study confirmed the extreme variability of the coralla of *D. dianthus* (solitary scleractinian; Fig. 1c, f, g, Fig. 1 in Addamo et al. [30]), higher than the variability of *L. pertusa*’s corallites (colonial scleractinian; Fig. 1a, b, d, e), consistent with the findings of previous studies of modern and Pleistocene samples [e.g., 20, 28, 30, 48]. The initial growth stages of the two taxa were hardly distinguishable, as it has been observed with other closely related caryophylliids [e.g. 49]. Coralla of larger juvenile *D. dianthus* (from 4 to 16 mm) were still very similar to *L. pertusa* corallites; however, at equal GCDs, the number of septa was higher in *D. dianthus* (see Additional file 1.4, Additional file 4; Fig. 1e, f) as confirmed by the Student’s two-tailed *t*-test (t-value is 12.107, *p*-value is <0.00001).

The main distinctive characters of the adult stages of *L. pertusa* and *D. dianthus* were a size maximum (Table 4) and growth form, colonial in the former species and solitary in the latter. Although some mass occurrences of *D. dianthus* looked like colonies (Fig. 1c), they were always formed by aggregation of coralla and not by budding as in *L. pertusa*. Indeed, individual polyps of aggregated *D. dianthus* were never internally connected, whereas the polyps of *L. pertusa* were always connected both externally and internally (Fig. 1b), at least during the early growth stage. Through growth, *L. pertusa* polyps can seal the connecting skeletal holes and, in some cases, occupy only the distal-most portion of the corallites, thus behaving as independent solitary scleractinians (e.g. Figure four a in [29]).

**Table 4:** Comparison of morphological characters observed in *D. dianthus* and *L. pertusa* ([20, 28] and reference therein, [30], this study)

**Characters**	***D. dianthus*** **(adult corallum)**	***L. pertusa*** **(adult corallite)**
Corallum/Corallite shape	Extremely variable. Typically trochoid with a subcylindrical pedicel, but also cylindrical, ceratoid, scolecoid	Variable, generally ceratoid, but also trochoid and subcilindrical; often curve.
Calyx shape	Elliptical to circular	Circular to slightly elliptical, often irregular
**Corallum/Corallite length (Max size)**	**L: up to 20 cm**	**L: exceptionally longer than 4 cm**
**Calicular diameter (Max size)**	**GCD: up to 9 cm**	**GCD: up to 2 cm**
Calicular fossa	Very narrow (Fw : LCD < 1:4) to large (1:3 < Fw : LCD < 1:2).Deep to very deep (Fd : LCD > > 2:3)	Generally narrow (1:4 < Fw : LCD < 1:3) to large (1:3 < Fw : LCD < 1:2). Deep to very deep (Fd : LCD > > 2:3)
Columella	Rudimental, visible only in juvenile specimens	Rudimental, visible only in juvenile specimens
Calicular margin	Flat to very jagged	Flat to very jagged
**Septa cycles**	**Up to 6 (incomplete)**	**Up to 4 (exceptionally few septa of cycle 5)**
Axial margin	Straight and continuous, undulated in the proximal zone (more evident in juvenile specimens)	Straight and continuous, undulated in the proximal zone
Septal granulation	Typically cone-shaped, secondarily subcylindrical, rarely hemispherical; decreasing in size and density from proximal to distal corallum; locally coalescing to form irregular septal ridges	Typically cone-shaped, secondarily subcylindrical, rarely hemispherical; decreasing in size and density from proximal to distal corallum; locally coalescing to form irregular septal ridges
**Tabulae**	**Rare**	**Common**
Costae	Typically acute (dominant septa) in the distalmost third of the corallum; rarely absent	Acute (dominant septa) in the distalmost fourth of the corallum or absent
Outer theca macro- and micromorphology	Diffuse conical to hemispherical granulation, seldom preferential along flat costae; denser and more raised in the basal part where furrows can be present	Diffuse granulation, seldom preferential along flat costae, denser and more raised in the proximal part
**Theca thickness : GCD**	**Variable; 0.01–0.36 (mostly 0.01–0.08)**	**Variable; 0.04–0.4 (mostly 0.06–0.14)**

Diagnostic characters clearly differentiating *D. dianthus* from *L. pertusa* (and vice versa) are indicated in bold

*Abbreviations*: *L* length, *GCD* greater calicular diameter, *LCD* lower calicular diameter, *F*_*w*_ width calicular fossa, *F*_*d*_ fossa depth

**Fig. 3:**
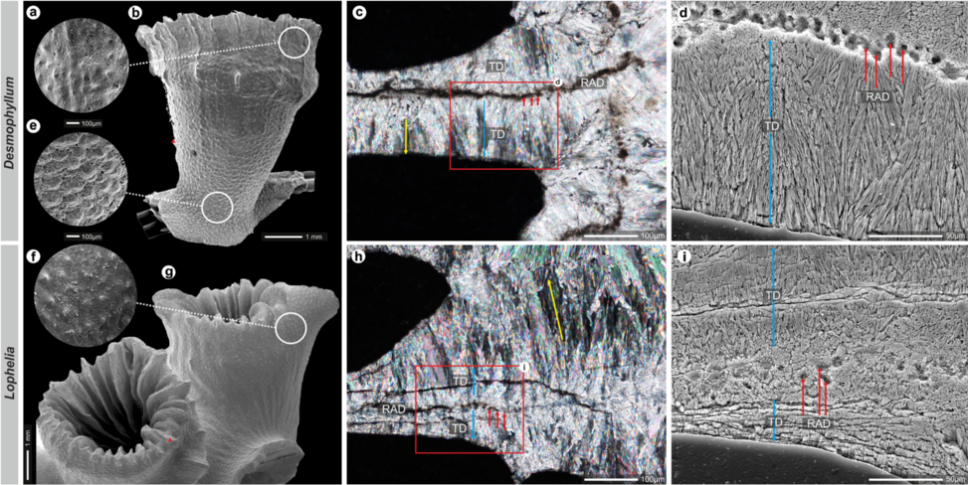
Micromorphology (thecal granulations) and microstructure of *Desmophyllum* *Dd*SML 188, **a**–**e** and *Lophelia* MEDCOR 09, **f**–**i**. Thecal granulations in both taxa are very similar: granules are typically denser and more rounded (hemispherical) in proximal portions of specimens (**d**) whereas more dispersed and spinose in distal portions (**a**, **e**). Microstructural organization of septa of both taxa as viewed in transverse thin sections (**c**, **h**) and polished and etched sections (**d**, **i**): the so called "mid-septal zone" consists of densely packed Rapid Accretion Deposits, RADs (red arrows) with Thickening Deposits, TD (blue arrows) radiating outward from the RADs. TD in the illustrated *Desmophyllum* form well organized, large bundles of fibers, whereas in *Lophelia*, few zones separated by clear crystal boundaries are recognizable. Yellow arrows (**c**, **h**) mark complete light extinction of the fiber bundles in polarized light, indicating similar arrangement of axes of individual crystallographic domains

Other macroscopic skeletal features were extremely variable between specimens of the same taxon and even changed within the same specimen (e.g. *L. pertusa* calice shape in Fig. 1a). Therefore, they could not be considered diagnostic taxonomic characters. Also, the skeletal microarchitecture of the two taxa was highly variable. The size and shape of both septal and outer thecal granulations tended to change with ontogenetic development and specimen size. In both *D. dianthus* and *L. pertusa,* granules were typically denser and larger (and often more rounded) in the proximal portions and more dispersed and smaller (and often more spinose) in distal portions (Fig. 3). This was particularly evident in large-sized specimens of *D. dianthus* in which portions of the distal corallum were often very smooth. However, the ratio of height basal diameter of the septal granules was highly variable, resulting in a wide spectrum of shapes ranging from hemispherical to subcylindrical and spinose.

At the microstructural level, *L. pertusa* and *D. dianthus* skeletons showed very similar organization. In both taxa, the septa and wall consisted of two distinct microstructural zones: the so-called "mid-septal zone" (or "dark line") composed of densely packed Rapid Accretion Deposits (RADs) (Fig. 3c, d, h, i) and Thickening Deposits (TD) composed of bundles of fibers that radiate outwards from the RADs. The spatial relationship between septal and wall microstructural units transformed similarly during ontogeny starting as marginotheca, evolving into trabeculotheca and eventually forming septotheca (see Additional file 5: red, blue and orange arrows, respectively, in vertical columns outlining). During the earliest ontogenetic stages, thecal RADs remained connected to RADs of the adjacent septa. In transverse sections of coralla, this was recognized as a continuation of the "dark line" between septa and wall. This microstructural pattern corresponded to the stage when the rims (consisting of RADs) of the wall and septa were formed at the same level. The theca, which was relatively thin during the initial phase (thus juvenile coralla have relatively thin bases), was covered from the outside by successive thickening deposits (called tectura, see [49]), resulting in very thick bases in adults. The next main ontogenetic phase started with the formation of the trabeculotheca. In transverse sections, this phase was recognized due to the interruption of the primary marginotheca by radial elements and the formation of trabeculothecal segments [see 49]. This microstructural pattern corresponded to the phase when the rims of (mainly) primary septa expanded above and outside of the wall rim, which consequently formed only as "bridges" between septa. The last ontogenetic phase of wall development started when trabeculothecal segments were no longer in interseptal zones, and the only existing wall was one formed of thickened portions of septa, i.e. septotheca. The septothecate stage, characterized by the increase in septal thickness, often occurred earlier in *L. pertusa* than in *D. dianthus* (see Additional file 5: b, h-d).

## Discussion

Mitochondrial gene order rearrangement and its phylogenetic implications have recently been reported for several genera of Scleractinia [15–18]. However, none of these studies described two morphologically distinct genera sharing an extremely high level of genetic similarity. The analysis of dN/dS ratios between the mitochondrial genomes of *L. pertusa* and *D. dianthus* indicates that sites are neutrally evolving. Of the approximately 16,000 bp per genome, more than 99 % were identical between these two taxa. With only 25 % of all differences identified as non-synonymous changes, any inferences on positive selection (or adaptation) would not be supported. Evidence of positive selection was detected for *L. pertusa* individuals from the Norwegian fjords and Mediterranean Sea, but without any evolutionary relevance [50]. Indeed, the positive selection found in this study (Table 2) was not statistically supported based on Fisher’s exact test due to the low number of substitutions in protein-coding regions. As remarked also by Flot et al. [50] more sensitive statistical procedures, such as the Z test, require at least 10 synonymous and 10 non-synonymous mutations for assumptions to be met [51]. In the case of *L. pertusa* individuals from the Norwegian fjords and Mediterranean Sea, adaptive evolution was inferred because only one (non-synonymous) substitution was detected in more than 16,000 bp. Given the absence of synonymous substitutions, the resulting dN/dS ratio was greater than 1, indicating positive selection. Therefore, the values obtained in this study could be an artefact resulting from the analysis.

Even if *Lophelia* and *Desmophyllum* are considered as well established distinct genera, the genetic similarity and a very close phylogenetic relationship between *L. pertusa* and *D. dianthus* have been previously suggested [24, 44, 47]. In these studies, several molecular markers were used, including mitochondrial and nuclear genes, non-coding and protein-coding genes (exons and introns) and markers with distinct variation levels (e.g. nucleotide sequences and microsatellite genotyping). Independent of the type of molecular marker used in this study, resulting genetic distances between these two genera were always less than 1 %, and in many cases, were equal to zero. In contrast, genetic divergence between *Desmophyllum* and other caryophylliid genera (excluding *Lophelia*) ranged from 2 to 7 % in both non-coding and protein-coding genes (see Additional file 1.5), showing a gene-dependent variation that correlates with the polymorphism level and mutation rate characterizing each marker. To date, a genetic threshold for clearly distinguishing species or genera has not been established, nor a consensus has been reached for defining a gene for universal DNA barcoding. The use of a genetic threshold or DNA barcoding is even more ambiguous if applied to Scleractinia, a taxonomic order characterized by slowing evolving mitogenomes and extensive interspecific hybridization [7, 52]. More than 1500 species of corals show a wide range of morphological variability and associated genetic incongruences at different phylogenetic levels [e.g. 53]. Furthermore, half of the scleractinian species live in the deep-sea, a more ‘stable’ habitat than tropical shallow waters, and therefore, are likely experiencing a different evolutionary rate.

The hypothesis that *Lophelia* and *Desmophyllum* have extremely slow evolution rates due to their preferential location in deep-water environments was tested using hypervariable genetic markers [24, 47]. Given the high mutation rates and level of polymorphisms, microsatellite markers are known to be powerful genetic tools for studying population structure. Microsatellites are usually designed to be species-specific markers; however, successful cross-species amplifications in related taxa are possible if the flanking regions of sequence repeats are conserved. Indeed, the evolutionary conservation of flanking regions of monomorphic microsatellite loci has been used as a source of data for resolving species-level phylogenies in several groups, including plants, fishes, and birds [see 54–56]. Therefore, two closely related species can be differentiated by the polymorphism of sequence repeats, exhibiting variance in nucleotide sequence or allele size (expressed in units of repeat counts). As previously demonstrated [47], 30 microsatellites markers, initially developed for *D. dianthus*, were successfully used, with clear peak profiles, to genotype *L. pertusa* specimens. Indeed, individuals of *L. pertusa* from the Mediterranean Sea and North Atlantic Ocean showed the same allele size range as *D. dianthus* (see Additional file 1.3, Additional file 3).

Nevertheless, similar microsatellite electromorphs can arise from independent mutational events, and such alleles can be not identical by descent [57]. Given this phenomenon, termed size homoplasy, additional comparative analyses using microsatellite sequences were performed. A total of 37 loci, previously characterized for *L. pertusa* [45, 58] and published in GenBank, were used to perform multiple BLAST searches using TRUFA 0.8.2 [59], against *D. dianthus* genomic DNA libraries that were previously obtained by Illumina (data not shown) and 454 [47] sequencing. This analysis of microsatellite sequences showed that average sequence identities for 1368 separate pair-wise comparisons between *L. pertusa* and *D. dianthus* were about 97 % similar*.*

Moreover, our data suggest that the putative mitochondrial control region may be a potential marker for investigating the phylogeography of the genera *Lophelia* [50] and *Desmophyllum*, though its usefulness in investigating species boundaries is uncertain. Interestingly, *D. dianthus* shows wide genetic divergence at the intraspecific level comparable to what is observed at the inter-generic level between *Desmophyllum* and *Lophelia* (Tables 1, 4, 2, see Additional file 1.1). Indeed, individuals that traditionally belong to the genera *Lophelia* and *Desmophyllum* are genetically more similar to each other than to individuals of the same genus (*Desmophyllum*) living in different regions (Chilean fjords vs Mediterranean Sea).

If 0.01–1 % genetic divergence in mitochondrial genomes, combined with morphological differences, is a valid range at the interspecific level for genera within Scleractinia (e.g. *Acropora divaricata* vs *Acropora aspera*, and *Porites okinawensis* vs *Porites panamensis*, see Additional file 1.2a and 1.2b), the results of our study suggest that Chilean *Desmophyllum "dianthus*", with its apparently morphological variation, and Mediterranean and Atlantic *L. pertusa* and *D. "dianthus"* may be considered three different species within the same genus. However, no clear morphological differences have been found between Mediterranean/Atlantic and Chilean *Desmophyllum* [54]; therefore the specific distinction should be only based on genetic information.

On the other hand, although statistical significance has been found between *D. dianthus* from *L. pertusa* for the number of septa (see Additional file 1, Additional file 4), the only unequivocal feature that allows us to distinguish the two taxa is asexual reproduction by budding in *L. pertusa*. Occasionally distinct *D. dianthus* polyps touch each other and fuse, producing a common skeleton, though no evidence of budding (in the polyp nor skeleton) has been reported for this species thus far. Therefore, the two growth forms, colonial (*L. pertusa*) and solitary (*D. dianthus*), can be morphologically distinguished when the first polyp reproduces asexually. The calice of *D. dianthus* can also reach much larger sizes than the one of *L. pertusa* (*L. pertusa* only rarely reach 20 mm in GCD) and, at equal GCD, almost always has a higher number of septa (see Additional file 1, Additional file 4). Moreover, though these two growth forms are commonly sympatric, in some cases, their relative dominance can be indicative of different environmental conditions, such as different habitats within the same area (e.g. Ionian Sea cold-water coral province [35, 36] or very distant geographic regions (e.g. *Lophelia*-dominated Norwegian fjords vs. *Desmophyllum*-dominated Chilean fjords). A clear distinction between the two growth forms is even easily detected in well-preserved fossil specimens recorded since the Early Miocene in peri-Mediterranean on-land outcrops [28, 60, 61].

The striking genetic similarity between *L. pertusa* and *D. dianthus* calls into question their assignment to two distinct genera, which was primarily based on growth form and secondarily on very few skeletal features. In order to preserve (palaeo) ecological information but, at the same time, update the scleractinian classification following new molecular results (as recommended by the Scleractinia Working Group (SWG) [62]), *D. dianthus* and *L. pertusa* should be ascribed at least to the same genus.

The genus *Desmophyllum* Ehrenberg, 1834 was described before the genus *Lophelia* (Milne Edwards & Haime, 1849). Thus, following the Principle of Priority of the International Code of Zoological Nomenclature (ICZN) [Art. 23.1] we propose that the solitary and the colonial species are ascribed herein to the genus *Desmophyllum* and are named *Desmophyllum dianthus* (Esper, 1794) and *Desmophyllum pertusum* (Linnaeus, 1758), respectively. Below we modify the diagnosis of the genus *Desmophyllum* based on the information acquired in this study. We also provide a short description of the most important skeletal features of the species *Desmophyllum pertusum* (Linnaeus, 1758)*.* A comprehensive revision of the genus *Desmophyllum*, including fossil species, is currently in progress.

Order: Scleractinia

[‘Robust’ Scleractinia Group]

Family: Caryophylliidae Dana, 1846

Genus: *Desmophyllum* Ehrenberg, 1834

**Diagnosis**: Corallum solitary or colonial. Corallum of solitary species greatly variable in shape, trochoid with subcylindrical pedicel or cylindrical, ceratoid, scolecoid. Colonial corallum bushy in shape, often with anastomosing branches. Up to 6 cycles of septa non-hexamerally arranged. Pali absent. Columella poorly developed, visible only in early developmental stages. Septothecal wall in ontogenetically adult corallites commonly covered by thickening deposits.

**Type species**: *Madrepora dianthus* Esper, 1794, pl. 69, Figures 1, 2, 3; subsequent designation Cairns, 1994, p. 76

Recent species included: *Desmophyllum striatum* Cairns, 1979—for description see Cairns (1979:120), *Desmophyllum quinarium* Tenison-Woods, 1879—for description see Tenison-Woods (1879:18), *Desmophyllum dianthus* (Esper, 1794)—for description see Cairns (1979:26), *Desmophyllum pertusum* (Linnaeus, 1758)

***Desmophyllum pertusum*** (Linnaeus, 1758) comb. nov.

*Madrepora pertusa* Linnaeus, 1758: 38, pl. 2, Figures 1, 2.

*Lophelia pertusa* Zibrowius, 1980: 126, pl. 66 a-l (synonymy).—Cairns, 2000: 100–102 (synonymy).

*Lophelia prolifera* Cairns, 1979: 125–127, pl. 24, Figures one, two, three, four, five (synonymy).

Diagnosis: Corallum forming large bushy colonies. Corallite skeleton connected to the parental one at least in the early growth stage (Fig. 1b). Corallites only exceptionally longer than 4 cm and up to 2 cm in maximum calicular diameter. Septa irregularly arranged in 4 cycles, only exceptionally in 5 incomplete ones. Common tabulae.

**Remarks**: The species has been widely described, as belonging to the genus *Lophelia* ([e.g., 20, 21, 27, 28, 63] and reference therein, [28]). The diagnosis reported herein includes only the skeletal characters that, at this stage of knowledge, lead us to distinguish *D. pertusum* from *D. dianthus*.

Insufficient information about *D. dianthus* and *D. pertusum* ecology and biology lead to be “conservative” in the taxonomic rearrangement. The difference in the geographical distribution, growth form (i.e. ontogenetic development), might be weak characters from a taxonomic point of view to maintain *D. dianthus* and *D. pertusum* as sympatric species. Detailed studies on the systematics of the genus *Desmophyllum,* currently on-going, might lead to synonymise these two species. Further research on biogeography and reproduction of the genus are needed to support (or not) this taxonomic distinction. If differences of environmental features and ontogenetic mechanism would not be statistically significant to support species differentiation, the comparison of molecular data performed in this study (including 5 complete mitochondrial genomes, 30 microsatellites, and 18 between protein and non protein-coding genes—Addamo, pers. obs.) would demonstrate that *D. dianthus* and *D. pertusum* are the same species. In such a case, following the rule of priority by date of publication of the International Code of Zoological Nomenclature (ICZN) [Art. 23.1], since the species *D. dianthus* (Esper, 1794) was described after *D. pertusum* (Linnaeus, 1758), both species would be named as *D. pertusum*.

Our findings pose questions about the molecular and developmental basis of colony formation in scleractinian corals and the taxonomic value of this character. Many lines of evidence show that in Cnidaria asexual budding and colony formation are controlled by differentially expressed genes. For example, Notch—a classical developmental signalling pathway, among other functions—is involved in asexual budding in *Hydra* (Hydrozoa), *Nematostella* (sea anemone), and *Acropora* (scleractinian) [see 64–66]. Taking into account the overwhelming genetic similarity between *Lophelia* and *Desmophyllum*, one may hypothesize that the same developmental mechanisms (i.e. Notch) regulate the solitary vs colonial growth forms of these taxa. There are several other examples of closely related scleractinians (genera, species) that display a variety of growth forms. For example, (1) *Anomocora carinata* includes both colonies *sensu stricto* (fully integrated) and “quasicolonial” forms in which “the daughter corallites [break] free of the parent before a third generation bud appears” [67], (2) *Rhizosmilia maculata* and *Dendrophyllia cornigera* show both colonies *s.s.* and "loosely integrated" colonies in which partial colonial mortality may yield solitary daughter polyps [20, 67], and (3) *Balanopsammia wirtzi* is represented by both solitary and partly to fully integrated colonies [68]. All these examples call into question the robustness of this widely used genus-level criterion, i.e. the occurrence of solitary vs colonial growth forms [69–71]. Further studies that recognize links between molecular and morphological characters [alike 25, 62, 72–74] and that focus on developmental (transcriptomic) mechanisms may help elucidate the basis of morphological and developmental mechanism variability and provide a robust taxonomic framework of Scleractinia.

## Conclusions

Molecularly and morphologically, solitary *Desmophyllum dianthus* and dendroid *Lophelia pertusa* appear to be significantly more similar to each other than other unambiguous coral genera analysed to date. Thus, following the ICZN, we propose to ascribe these two species to the genus *Desmophyllum* and to name them as *D. dianthus* and *D. pertusum*. Findings of this study may have broader implications that should lead to re-consider the taxonomic value of growth forms (solitary vs colonial), traditionally used to distinguish scleractinian genera. Further integrative studies combining molecular, developmental biology and ecological environmental studies are required to test the potential conspecificity of *D. dianthus* and *D. pertusum* and provide more insights into the evolution of Scleractinia.

## Methods

### Sample collection and study area

The coral specimens selected for genetic analysis were collected during several oceanographic expeditions (Additional file 1.6) authorized by the competent maritime authorities off-shore Italy, Ireland, Argentina, Chile, Tasmania and Australia. The CORSARO 39 and MEMA12 cruises were carried out in the frame of EU projects Hermes (GOCE-CT-2005-511234-1) and FP7 Hermione (grant agreement no: 226354). The Eurofleets Moira Mound cruise [75] was funded by the European Union Seventh Framework Programme (EU-FP7/2007–2013) under the Eurofleets grant agreement n. 228344. The expedition in the Chilean fjords was performed in the frame of Spanish project funded by Spanish Research Council (CSIC) and Endesa Foundation.

Some of the coral specimens used for skeletal analysis (Additional file 1.4) were genetically analysed by [24]. The other specimens were collected dead 1) off-shore Ireland during the Eurofleets Moira Mound cruise (see above), 2) off-shore Morocco (during the Genesis 2 and MD194 [76, 77] cruises, framed within the EU-FP7 Hermione and Eurofleets projects (see above), respectively) and 3) off-shore Italy (during the MAGIC-CoralFISH and METEOR 70-1 cruises). The Magic-CoralFISH cruise [78] was funded by the EU-FP7 CoralFISH Project (grant agreement n. 213144) and the MAGIC Project by the Italian National Research Council. Details about the METEOR 70-1 cruise can be found in [33].

After collection, all specimens were preserved in absolute ethanol. Samples were transported to Spain with appropriate export and import permits following the Convention on International Trade in Endangered Species of wild Fauna and Flora (CITES). This study did not involve endangered or protected species listed in the IUCN Red List of Threatened Species.

#### Genetic analysis

For amplification of the complete mitochondrial genome, two samples of *D. dianthus* were collected in two distant localities: 1) South Adriatic Sea (39°53’468”N, 18°55’176”E), off shore of Tricase (Italy, Mediterranean Sea), sampled at a depth of 786 m by the R/V *Urania* during CNR cruise MEMA12 during April to May 2012 and 2) Isla Jaime (43°46’34.23”S, 72°55’13.057”W), located in the Pitipalena Fjord (Chile, South Pacific Ocean), sampled at a depth of 23 m by SCUBA diving in February 2012.

For additional sequence comparisons of the putative control region, 7 samples of *D. dianthus* and 2 of *L. pertusa* were collected in 10 distinct localities distributed in the northern and southern hemispheres. Specimen information is found in Additional file 1.6.

### DNA extraction and mitochondrial genome sequencing

Genomic DNA was extracted from the mesenteric tissue of each specimen using the QIAGEN BioSprint 15 DNA Blood Kit (Qiagen Iberia S.L., Madrid, Spain), with slight modifications, including the optional RNase treatment and an extended period of proteinase K lysis (overnight incubation at 55 °C). The DNA was quantified using a Qubit 2.0 Fluorometer and diluted to a final concentration of 2 ng/μl.

Several overlapping fragments, covering the entire mitogenome, were amplified by PCR mainly using primers previously designed for *L. pertusa* [50], though one specific primer pair was designed using PRIMER3 [79] (Table 5). PCRs were carried out in a total volume of 50 μl with 1x PCR Biotools Standard Reaction Buffer including 2 mM MgCl_2_, 0.5 μM forward and reverse primers, 0.2 mM of each dNTP, 1.5U DNA polymerase (Biotools), and 2 ng of template DNA. PCR amplifications were performed in a Veriti™ Thermal Cycler (Applied Biosystems) with the following cycle conditions: an initial denaturing step of 94 °C for 5 min, followed by 35 cycles of 30 s at 94 °C, an annealing step of 30 s at 53 °C, an extension step of 1–3 min at 72 °C, and a final extension of 10 min at 72 °C. PCR products were purified using GELase™ Agarose Gel-Digesting Preparation (Epicentre, Madison, WI, USA), following the Fast Protocol. If a specific PCR product was not amplified under these conditions, three other annealing temperatures (T_A_ 48, 50, or 51 °C) were tested with the same cycling conditions. Failing that, PCR amplifications were carried out as above but in a total volume of 20 μl and with 2U DNA polymerase (MyTaq). In these cases, PCR amplifications were performed with the following cycle conditions: an initial denaturing step at 95 °C for 5 min, followed by 40 cycles of 15 s at 95 °C, an annealing step of 30 s at 50 °C, an extension step of 1–3 min at 72 °C, and a final extension of 10 min at 72 °C.

**Table 5:** Primers pairs used for amplification and sequencing

Oligo name	Oligo sequence (5' to 3')	Fragment length (bp)	Reference
LD1F	AAATCAAACGAGATTCCGAGAG	1198	Flot et al. 2013 [50]
LD1R	TCCATGGGGACTTCTCGTC	–	Flot et al. 2013 [50]
LD2F	TCGACTGTTTACCAAAAACATAGC	1519	Flot et al. 2013 [50]
LD2R	AAYAACCTTCCATTGCATCC	–	Flot et al. 2013 [50]
LD3F	TAGGAGTGGTTGGGAAATCG	2563	Flot et al. 2013 [50]
LD3R	CTTGGGGAAGCCAAATATGA	–	Flot et al. 2013 [50]
LD4F	GAACAACAGGGGCAACAGAT	2127	Flot et al. 2013 [50]
LD4R	ATGGTGTCCCTGAAAAGTCG	–	Flot et al. 2013 [50]
LD5F	GCAGACGCGGTGAAACTTA	2521	Flot et al. 2013 [50]
LD5R	TACCCCGGCTAAGACAACTG	–	Flot et al. 2013 [50]
LD6F	TTGTGGGGCAAATCATTCTT	1034	Flot et al. 2013 [50]
LD6R	AATGAGAAAGCCCACAAGCA	–	Flot et al. 2013 [50]
LD7F	CAACTCCGGTTTCTGCCTTA	3060	Flot et al. 2013 [50]
LD7R	TTTAAAAGAAAACTATGGAGGCCTAA	–	Flot et al. 2013 [50]
LD8F	TTATTGGGCCTGTGTTTGGT	1604	Flot et al. 2013 [50]
LD8R	CCCACATATGAAAAGGAGCAAC	–	Flot et al. 2013 [50]
LD9F	TGGGTGCTCTTTCTTCTGGT	1237	Flot et al. 2013 [50]
LD9R	AAATCCAATTGGTATATAATTTGTCA	–	Flot et al. 2013 [50]
LD10F	ATCCCTCCTTTTGCAGGATT	868	Flot et al. 2013 [50]
LD10R	CCCCAGAAGCTGTTGTGTTT	–	Flot et al. 2013 [50]
LD11F	GGCAATTGGTTCTGGGATAA	1254	Flot et al. 2013 [50]
LD11R	AAGCATACTAAAAGCCGTTCCA	–	Flot et al. 2013 [50]
LD12F	TCTACAAACCACAAAGATATCGG	930	This study
LD12R	AATCCCCGTAGGAACAGCAA	–	This study
LD13F	GCCGGTGCTATTACAATGCT	1892	Flot et al. 2013 [50]
LD13R	CAATCGATTCAAGCTCTTTTCA	–	Flot et al. 2013 [50]
1a.PWF	CCATGTCCCACGGTTTATGT	–	This study
1b.PWR	AGGCCCAACTAACCTTCCAT	–	This study
2a.PWF	CATGGCGATTTCTTCTGTGA	–	This study
2b.PWR	CCCCGTCACACTTATGATCC	–	This study
3.PWF	GAAGCTTTTGTCATGCTTCCTT	–	This study
4a.PWF	TGTGGAGTTTTCTCCTTGACC	–	This study
4b.PWR	AAGCTAACGTCTCGCCTTCA	–	This study
5a.PWF	GGTTGTGGCTTGTGGTCTTT	–	This study
5b.PWR	GCCCTCAAGGCAAAACATAA	–	This study
6a.PWF	ACAGTCGGGGCAAGTTTTTA	–	This study
6b.PWR	ACCAAACACAGGCCCAATAA	–	This study

*Abbreviations*: *PW* primer walking

To amplify the putative control region, PCR amplifications were performed in total volume of 20 μl with 1x PCR OptiBuffer Reaction Buffer including 3 mM MgCl_2_, 1x Hi-Spec Additive, 0.5 μM forward and reverse primers, 0.5 mM of each dNTP, 2U DNA polymerase (BIO-X-ACT Short), and 2 ng of template DNA. PCR amplifications were performed with the following cycle conditions: an initial denaturing step at 95 °C for 5 min, followed by 30 cycles of 30 s at 94 °C, an annealing step of 30 s at 56 °C, an extension step of 2 min at 72 °C, and a final extension of 10 min at 72 °C.

Individual amplicons were cloned into pGEM-T vectors (Promega, Madison, WI, USA), were purified using the Wizard^®^*Plus* SV Minipreps DNA Purification System (Promega, Madison, WI, USA), following the Centrifugation Protocol, and sequenced on ABI PRISM 3730 DNA Sequencer (Applied Biosystems), following the Poly-A/T Protocol (Secugen S.L.) using specific primers, M13 universal primers and, if necessary, internal (walking) primers to cover the total length of the fragments (Table 5).

The complete mitogenomes and sequences reported in this paper were deposited in GenBank (NCBI).

### Sequences alignment, annotation and analyses

Sequence chromatograms were verified, and primer sequences removed using Sequencher v.4.10.1 (Gene-Code Corporation). Genomic sequences were confirmed using BLAST (NCBI), assembled using Sequencher v4.10.1, and then compared with three previously published *L. pertusa* mitogenomes [15, 50] (see Additional file 1.7).

Open reading frames (ORFs) were identified using ORF Finder (available online at http://www.bioinformatics.org/sms2/orf_find.html), with search parameters set to codon length > 50 amino acids and the Coelenterate Mitochondrial Code translation. Transfer RNA genes were identified using tRNAscan-SE 1.21 [80] (available online at http://lowelab.ucsc.edu/tRNAscan-SE/). Additional automatic annotations were performed with DOGMA [81] (available online at http://dogma.ccbb.utexas.edu/), using high COVE threshold for mitochondrial tRNAs (=30) and MITOS [82] (available online at http://mitos.bioinf.uni-leipzig.de/index.py). The mitochondrial protein-coding genes were compared to calculate non-synonymous (dN) and synonymous (dS) substitution rates through model selection and model averaging using three different methods based on Maximum-Likelihood, implemented in KaKs_Calculator [83].

To compare the genomes of a larger range of species, 50 previously published coral mitogenomes, representing 5 families and 15 genera of the Scleractinia Order, were retrieved from GenBank and aligned in ClustalW [84], using the default settings (see Additional file 1.7). The resulting alignments were manually checked and adjusted with Se-Al v.2.0a11 [85]. Estimation of genetic divergence between pairs of taxa, using uncorrected p-distances, were calculated in PAUP*v4.0a134 [86]. To estimate genetic divergence among genera and families of corals, mean uncorrected p-distances were calculated in Sequencer 6.1 (shareware written by B. Kessing).

Additional comparative analyses were performed using previously characterized microsatellite sequences for *L. pertusa* (37 loci [45, 58]) and *D. dianthus* (30 loci [47, 87]). Multiple BLAST searches of the genomic DNA libraries of both species were performed using TRUFA 0.8.2 [59].

#### Skeletal analysis

The main differences and similarities between coralla and corallites of *D. dianthus* and *L. pertusa*, respectively (Table 4), were identified based on observations of 200 selected specimens (see Additional file 1.4) and data from the literature [20, 27, 41, 48]. To assess comparable skeletal features of *D. dianthus* and *L. pertusa,* individuals were selected based on having a Greater Calicular Diameter (GCD) between 4 and 16 mm. This GCD range is a relevant parameter for the following reasons: 1) both species have sizes within this GCD range; 2) *L. pertusa* rarely has corallites with a GCD > 16 mm; 3) corallites with a GCD <4 mm are indistinguishable between *D. dianthus* and *L. pertusa* species, and among other caryophylliids. The normality of number of septa distribution has been tested for each species and, once it was confirmed, a Student’s two-tailed *t*-test has been performed for each parameter for both species. Analyses of septal and outer wall granulations were carried out on specimens selected for genetic analyses (see Additional file 1.4). Skeletal micromorphology was examined by SEM: specimens were mounted using silver glue, sputter-coated with conductive gold or platinum and analysed using a Vega Tescan (University of Milano-Bicocca) or Phillips XL20 (Institute of Paleobiology, Warsaw) scanning electron microscope. Microstructural and ontogenetic data were obtained from serial transverse thin sections of coralla observed under a conventional transmitted light microscope. Polished sections were examined using a Nikon Eclipse 80i transmitted light microscope fitted with a DS-5Mc cooled camera head (Institute of Paleobiology, Warsaw). Supplementary ontogenetic micro-CT data were collected with a Zeiss XRadia MicroXCT-200 system equipped with a 90 kV/8 W tungsten X-ray source at the Laboratory of Microtomography, Institute of Paleobiology, Warsaw. Scans were performed using the following parameters: voltage = 60 kV, power = 8 W, pixel size ca. 21 μm, 1601 projections per sample, exposure time 4 s. Radial projections were reconstructed with XMReconstructor software. The major changes in ontogenetic development were described following an ontogenetic sequence of thecal structures proposed by Stolarski [49]. Skeletal microstructural terminologies, namely the recognition of two main microstructural components, Rapid Accretion Deposits (RAD; also called Centers of Calcification) and Thickening Deposits (TD; also called fibers), were used according to Stolarski [88].

## Ethics approval and consent to participate

Not applicable.

## Consent for publication

Not applicable.

## Availability of supporting data

The DNA sequences supporting of this article are available in [GenBank] database [Accession Numbers KX000882-KX000894].

The new taxon name and this article are registered in [ZooBank] registry with an identifier code [urn:lsid:zoobank.org:act:949C5CE4-4841-423 F-958D-A8D6A4E65E6B].

## Additional files

Additional file 1: 1.1 Annotation of the complete mitochondrial genome of *D. dianthus* (Dd) and *L. pertusa* (Lp) using DOGMA (Wayman et al. 2014) [77]. **1.2** Genetic divergences among scleractinian species. **a.** Genetic divergences among mitochondrial genomes of scleractinian species. **b.** Minimum (MIN) and maximum (MAX) value of genetic divergences (in percentage) among mitochondrial genomes of scleractinian species at inter—and intrageneric taxonomic level. **1.3** Comparison of *D. dianthus* and *L. pertusa* allele sizes for 30 microsatellite loci [47]. **1.4** Measurements of Great Calicular Diameter (GCD) and septa number (Septa N.) for 200 specimens of *D. dianthus* and *L. pertusa*. **1.5** Genetic divergence between *D. dianthus* (*Dd*) and *L. pertusa* (*Lp*), or other *Dd* individuals, *Caryophyllia* species (*C. spp*), or other hexacorallian species (Outgroup) [24]. **1.6** Depth, technique, geographic coordinates and other information for samples analysed in this study. **1.7** List of mitochondrial genomes analysed in this study with associated GenBank accession numbers (updated July 2014). (XLSX 53 kb) Additional file 2: Synonymous and non-synonymous substitutions within the mitochondrial genomes of three individuals of *L. pertusa* and two of *D. dianthus *. (PDF 75 kb) Additional file 3: Plots showing the allele size frequency per loci of *D. dianthus* (blue) and *L. pertusa* (red) for the 30 new microsatellites markers developed and described in Addamo et al. [47]. (PDF 2595 kb) Additional file 4: Plot showing the relationship between the Greater Calicular Diameter (GCD in mm) and number of septa (S) in juvenile coralla of *D. dianthus* (blue diamonds) and *L. pertusa* (red diamonds). At equal GCD, the number of septa is normally higher in *D. dianthus* than in *L. pertusa*; there is also larger variability in septal number at a given GCD in *D. dianthus* than *L. pertusa*. (PDF 61 kb) Additional file 5: Ontogenetic and microstructural skeletal features of *Desmophyllum dianthus* (Dd ROC 180, a, e, i, m, r, v—virtual mCT sections; b, f, j, n, s, w—thin sections, transmitted light microscope) and *Lophelia pertusa* (MEDCOR 09, c, g, k, o, t, z—virtual mCT sections; d, h, l, p, u, x—thin sections, transmitted light microscope). In both taxa, spatial relationships between the septa and wall transform similarly during ontogeny, described as a thecal sequence from marginotheca (red arrows) to trabeculotheca (blue arrows) to septotheca (orange arrows in vertical columns). Virtual mCT sections (left column for each taxon) correspond to the phases of growth depicted in transverse thin sections (right column for each taxon). Septotheca typically develops slightly later in the ontogeny of *Desmophyllum* (see length of orange arrows). Microstructural organization of coralla of both taxa is very similar and simple: septa and wall consist of densely packed Rapid Accretion Deposits, RAD (traditional "centers of calcification") and Thickening Deposits, TD (traditional fibers), which radiate outward (in transverse sections) from RADs. (PDF 12352 kb)
