# Erratum to: Merging scleractinian genera: the overwhelming genetic similarity between solitary *Desmophyllum* and colonial *Lophelia*

**DOI:** 10.1186/s12862-016-0703-3

**Published:** 2016-07-19

**Authors:** A. M. Addamo, A. Vertino, J. Stolarski, R. García-Jiménez, M. Taviani, A. Machordom

**Affiliations:** Departamento de Biodiversidad y Biología Evolutiva, Museo Nacional de Ciencias Naturales (MNCN-CSIC), José Gutiérrez Abascal 2, Madrid, 28006 Spain; Dipartimento di Scienze dell’Ambiente e del Territorio e di Scienze della Terra, Università di Milano Bicocca (UNIMIB), Piazza della Scienza 4, Milan, 20126 Italy; Department of Geology Renard Centre of Marine Geology, Universiteit Ghent, Krijgslaan 281, Ghent, B-9000 Belgium; Polskiej Akademii Nauk, Instytut Paleobiologii, Twarda 51/55, Warsaw, PL-00-818 Poland; Consiglio Nazionale delle Ricerche, Istituto di Scienze Marine (ISMAR), Via Gobetti 101, Bologna, 40129 Italy; Biology Department, Woods Hole Oceanographic Institution, 266 Woods Hole Road, Woods Hole, MA 02543 USA; Stazione Zoologica Anton Dohrn, Villa Comunale, Naples, 80121 Italy

## Erratum

As a result of vendor errors being introduced during processing, the original version of this article [1] was published with some duplication errors in Table 1.

**Table 1:** Pairwise species non-synonymous substitutions with nucleotide (NT) and amino acid (AA) location

				*Dd*432-*Lp*KC875348				*Dd*432-*Lp*KC875349				*Dd*432-*Lp*FR821799				*Dd*432-*Dd*636				*Dd*636-*Lp*KC875348				*Dd*636-*Lp*KC875349				*Dd*636-*Lp*FR821799			
#	AA	NT	Gene	AA				AA				AA				AA				AA				AA				AA			
1	26	77	*nad5*	26	R	==>	K	26	R	==>	K	26	R	==>	K					26	R	==>	K	26	R	==>	K	26	R	==>	K
2	38	113	*nad5*	38	T	==>	I	38	T	==>	I	38	T	==>	I	38	T	==>	I												
3	165	493	*nad5*	165	V	==>	I	165	V	==>	I	165	V	==>	I	165	V	==>	I												
4	351	1051	*nad1*													351	K	==>	Q	351	Q	==>	K	351	Q	==>	K	351	Q	==>	K
5	427	1280	*nad1*	427	C	==>	F	427	C	==>	F					427	C	==>	F									427	F	==>	C
6	1403	4208	*cox3*													1403	N	==>	S	1403	S	==>	N	1403	S	==>	N	1403	S	==>	N
7	1408	4225	*cox3*	1408	G	==>	S	1408	G	==>	S	1408	G	==>	S	1409	G	==>	S												
8	1499	4498	*cox3*	1499	S	==>	G	1499	S	==>	G	1499	S	==>	G					1500	S	==>	G	1500	S	==>	G	1500	S	==>	G
9	1685	5056	*cox2*	1685	G	==>	R	1685	G	==>	R	1685	G	==>	R	1686	G	==>	R												
10	2289	6865	*nad5*									2288	E	==>	K													2289	E	==>	K
11	2471	7411	*cob*									2470	T	==>	P													2471	T	==>	P
12	2698	8097	*cob*	2698	L	==>	F	2698	L	==>	F									2699	L	==>	F	2699	L	==>	F				
13	2863	8589	*nad2*													2863	F	==>	L	2863	L	==>	F	2863	L	==>	F	2863	L	==>	F
14	2930	8789	*nad2*													2930	H	==>	R	2930	R	==>	H	2930	R	==>	H	2930	R	==>	H
15	3054	9161	*nad2*									3053	L	==>	S													3054	L	==>	S
16	3087	9260	*nad6*									3086	L	==>	P													3087	L	==>	P
17	3191	9574	*nad6*	3191	Y	==>	H													3192	Y	==>	H								
18	3221	9664	*nad6*	3221	L	==>	I	3221	L	==>	I	3221	L	==>	I					3222	L	==>	I	3222	L	==>	I	3222	L	==>	I
19	3348	10046	*cox1*	3348	A	==>	V	3348	A	==>	V	3348	A	==>	V					3349	A	==>	V	3349	A	==>	V	3349	A	==>	V
20	3354	10064	*cox1*	3354	V	==>	A	3354	V	==>	A	3354	V	==>	A					3355	V	==>	A	3355	V	==>	A	3355	V	==>	A
21	3389	10166	*cox1*									3388	S	==>	F													3389	S	==>	F
22	3713	11141	*cox1*	3713	R	==>	K	3713	R	==>	K	3713	R	==>	K					3714	R	==>	K	3714	R	==>	K				

*Dd* Desmoplyllum dianthus, *Lp* Lophelia pertusa

Dd432-LpKC875348 Dd432-LpKC875349 Dd432-LpFR821799 Dd432Dd636Dd432Dd636 Dd636LpKC875348Dd636LpKC875348 Dd636LpKC875348Dd636LpKC875348 Dd636LpFR821799Dd636LpFR821799

Should be:

Dd432-LpKC875348 Dd432-LpKC875349 Dd432-LpFR821799 Dd432-Dd636 Dd636-LpKC875348 Dd636-LpKC875349 Dd636-LpFR821799

In addition, the alignment of Table 1 attribute listed as “AA” is incorrect and should correspond as shown in the below updated version.

Lastly Page 10, third paragraph included an incorrect citation. This should read as “(see Additional file 1.4, Additional file 4)” instead of “(see Additional file 1, Additional file 4)”.

The original version of this manuscript has been updated to reflect the above changes.

We apologise for any inconvenience this has caused.
