# Supplementary material for: Merging scleractinian genera: the overwhelming genetic similarity between solitary Desmophyllum and colonial Lophelia
Source: BMC Evol Biol. 2016 May 18;16:108. doi: 10.1186/s12862-016-0654-8 (PMC4870751; doi:10.1186/s12862-016-0654-8)
Supplement: Additional file 2: — Synonymous and non-synonymous substitutions within the mitochondrial genomes of three individuals of L. pertusa and two of D. dianthus . (PDF 75 kb) [file 12862_2016_654_MOESM2_ESM.pdf]

```
[            1         11        21        31        41        51        61        71        81        91        ]
[            |         |         |         |         |         |         |         |         |         |         ]
Dd636        ATGTATCTTTTAGTTTTGTTTTTCCCTTTAATTGGGGCGGTTTTAGTTGGTTCTTTTGGACGAAAAATAGGGGAAAGGGGTGCTGGTGTTTTAACTTCCT
Dd432        ....................................................................................................
Lp_KC875348  ............................................................................A.......................
Lp_KC875349  ............................................................................A.......................
Lp_FR821799  ............................................................................A.......................
ChangesNS    ............................................................................N.......................

[            101       111       121       131       141       151       161       171       181       191       ]
[            |         |         |         |         |         |         |         |         |         |         ]
Dd636        GTTGTTTAATTATAAGTCTTTCTTATTCTGTTTTTATTGGGGCAGAAATTTTATTTAACTCAACAATAACATATATTAAGTTGTGAAAGTGATTTGATTC
Dd432        ............C.......................................................................................
Lp_KC875348  ....................................................................................................
Lp_KC875349  ....................................................................................................
Lp_FR821799  ....................................................................................................
ChangesNS    ............S.......................................................................................

[            201       211       221       231       241       251       261       271       281       291       ]
[            |         |         |         |         |         |         |         |         |         |         ]
Dd636        AGGATCTTTCCTTGTTTTTTTTGGTTTACAATTTGATTGTTTAGTTGTGATTATGTTGTTTGTTGTTTTTATTGTTTCTACTTTGGTTCATATTTTTTCT
Dd432        ....................................................................................................
Lp_KC875348  ....................................................................................................
Lp_KC875349  ....................................................................................................
Lp_FR821799  ....................................................................................................
ChangesNS    ....................................................................................................

[            301       311       321       331       341       351       361       371       381       391       ]
[            |         |         |         |         |         |         |         |         |         |         ]
Dd636        ATTGCCTACATGCGAGGAGATCCCCATGTCCCACGGTTTATGTCTTATCTTTCTTTGTTTACTTTTTTAATGGTTGTTTTAGTAACAAGTGATAATTTCC
Dd432        ........T...........................................................................................
Lp_KC875348  ....................................................................................................
Lp_KC875349  ....................................................................................................
Lp_FR821799  ....................................................................................................
ChangesNS    ........S...........................................................................................

[            401       411       421       431       441       451       461       471       481       491       ]
[            |         |         |         |         |         |         |         |         |         |         ]
Dd636        TTCAGTTATTTATTGGCTGGGAGGGGGTAGGCCTTTGTTCTTATTTATTAATTAATTTTTGATTAACAAGATTAGAAGCAAACAGAGCTGCGATTAAAGC
Dd432        ............................................................................................G.......
Lp_KC875348  ....................................................................................................
Lp_KC875349  ....................................................................................................
Lp_FR821799  ....................................................................................................
ChangesNS    ............................................................................................N.......
```

```
[           501       511       521       531       541       551       561       571       581       591        ]
[           |         |         |         |         |         |         |         |         |         |         ]
Dd636       AATGTTAGTAAATCGAGTGGGGGACATTGGGTTGCTTTTAGCAATGTTTCTTATTTGAAATGTTTTTGGGACCTTAGACTTTTCTTCTGTTTTTAATTTA
Dd432       ....................................................................................................
Lp_KC875348 ....................................................................................................
Lp_KC875349 ....................................................................................................
Lp_FR821799 ....................................................................................................
ChangesNS   ....................................................................................................

[           601       611       621       631       641       651       661       671       681       691        ]
[           |         |         |         |         |         |         |         |         |         |         ]
Dd636       GTTTTTTGTTGTTCTGATCAAATTTTTTTTATTTGTTTATTCTTGTTCTTAGGAGTGGTTGGTAAATCGGCTCAATTGGGGCTACACACTTGATTACCGG
Dd432       ..............................................................G.....................................
Lp_KC875348 ..............................................................G.....................................
Lp_KC875349 ..............................................................G.....................................
Lp_FR821799 ..............................................................G.....................................
ChangesNS   ..............................................................S.....................................

[           701       711       721       731       741       751       761       771       781       791        ]
[           |         |         |         |         |         |         |         |         |         |         ]
Dd636       ATGCAATGGAAGGTTAGTTGGGCCTTTTAATTAAAAAATTAATTAAAAACGCCACTATGATTATAAAAGCTTTCGTTGTTCTTGTCCCTTTACTTATTGC
Dd432       .........................................................................T..........................
Lp_KC875348 .........................................................................T..........................
Lp_KC875349 .........................................................................T..........................
Lp_FR821799 .........................................................................T..........................
ChangesNS   .........................................................................S..........................

[           801       811       821       831       841       851       861       871       881       891        ]
[           |         |         |         |         |         |         |         |         |         |         ]
Dd636       TGTGGCATATTTAACTTTAGCAGAACGAAAGGTTTTAGGGTACATGCAAGCAAGAAAAGGACCTAATGTGGTTGGGGGGGGNTTGCTTCAGCCTTTTGCG
Dd432       .................................................................................G..................
Lp_KC875348 .................................................................................G..................
Lp_KC875349 .................................................................................G..................
Lp_FR821799 .................................................................................G..................
ChangesNS   .................................................................................S..................

[           901       911       921       931       941       951       961       971       981       991        ]
[           |         |         |         |         |         |         |         |         |         |         ]
Dd636       GATGGGATTAAGTTATTTCTTAAAGAAATGGTTATTCCCCATCGAGTGAGTGGGTTTGTTTATCTTTTAGCCCCAGTTCTTTCTTTTATTTTGGCTTTTA
Dd432       ....................................................................................................
Lp_KC875348 ....................................................................................................
Lp_KC875349 ....................................................................................................
Lp_FR821799 ....................................................................................................
ChangesNS   ....................................................................................................
```

```
[            1001      1011      1021      1031      1041      1051      1061      1071      1081      1091      ]
[            |         |         |         |         |         |         |         |         |         |         ]
Dd636        TTGTTTGGGGTATCCTCCCTTATAATAAGGGGGTTGGAATAAGTGATTTTCAAATTGGTCTTTTATGAATCATGGCGATTTCTTCTGTGAGTGTTTATGC
Dd432        ..................................................A.................................................
Lp_KC875348  .............T....................................A.................................................
Lp_KC875349  .............T....................................A.................................................
Lp_FR821799  .............T....................................A.................................................
ChangesNS    .............S....................................N.................................................

[            1101      1111      1121      1131      1141      1151      1161      1171      1181      1191      ]
[            |         |         |         |         |         |         |         |         |         |         ]
Dd636        TATTTTAATGTCGGGCTGAGGCAGTCGTTCTAAATATGCTTTTTTAGGTGCGGTCCGAGCTGCGGCGCAAATGATTAGTTATGAGGTTTCTATTGGTTTA
Dd432        ....................................................................................................
Lp_KC875348  ....................................................................................................
Lp_KC875349  ....................................................................................................
Lp_FR821799  ....................................................................................................
ChangesNS    ....................................................................................................

[            1201      1211      1221      1231      1241      1251      1261      1271      1281      1291      ]
[            |         |         |         |         |         |         |         |         |         |         ]
Dd636        ATTATTATTTCTGTTCTTTTATGTGTTGGGTCTTTGTCTTTAAATGAAATTGTATTAACACAAAATGAAATTTGGTTTTTTTTTCCCCTTTTTCCTGTTG
Dd432        ...............................................................................G....................
Lp_KC875348  ....................................................................................................
Lp_KC875349  ....................................................................................................
Lp_FR821799  ...............................................................................G....................
ChangesNS    ...............................................................................N....................

[            1301      1311      1321      1331      1341      1351      1361      1371      1381      1391      ]
[            |         |         |         |         |         |         |         |         |         |         ]
Dd636        TTATAATGTTWCTCGTTTCTATTTTAGCAGAAACAAACCGTGCTCCCTTTGATTTAACARAAGGAGAGTCGGAGCTRGTGTCGGGGTACAACGTAGAGTA
Dd432        ..........T................................................G................A.......................
Lp_KC875348  ..........T................................................G................A.......................
Lp_KC875349  ..........T................................................G................A.......................
Lp_FR821799  ..........T................................................G................A.......................
ChangesNS    ..........S................................................S................S.......................

[            1401      1411      1421      1431      1441      1451      1461      1471      1481      1491      ]
[            |         |         |         |         |         |         |         |         |         |         ]
Dd636        CGCTTCGATGTCTTTTGCTCTATTTTTTCTTGCCGAATATGCTCATATTATATTTATGAGTTGTTTAACTGTTCTTTTATTTTTTGGAGGATGGTTGCTT
Dd432        ....................................................................................................
Lp_KC875348  ....................................................................................................
Lp_KC875349  ....................................................................................................
Lp_FR821799  ....................................................................................................
ChangesNS    ....................................................................................................
```

```
[             1501      1511      1521      1531      1541      1551      1561      1571      1581      1591       ]
[             |         |         |         |         |         |         |         |         |         |          ]
Dd636         TTGCCCCTTGGTTTTAAAACCGTTTTTATTGTTTTATTTTTTTTATGGGCACGGGCCTCTTTCCCTAGGGTCCGGTATGATCAATTGATGGYCCTATTAT
Dd432         ...........................................................................................C........
Lp_KC875348   ...........................................................................................C........
Lp_KC875349   ...........................................................................................C........
Lp_FR821799   ...........................................................................................C........
ChangesNS     ...........................................................................................S........

[             1601      1611      1621      1631      1641      1651      1661      1671      1681      1691       ]
[             |         |         |         |         |         |         |         |         |         |          ]
Dd636         GAAAGGGGTATTTACCTTTAAGTTTAGGGATTGTTATTTTTGTTGCCAGTATTTTATTCGGGTTTAATGGTTCTCCTCCG---ATGAGTGGTGCTTATTT
Dd432         ................................................................................ATT.................
Lp_KC875348   ................................................................................ATT.................
Lp_KC875349   ................................................................................ATT.................
Lp_FR821799   ................................................................................ATT.................
ChangesNS     ................................................................................---.................

[             1701      1711      1721      1731      1741      1751      1761      1771      1781      1791       ]
[             |         |         |         |         |         |         |         |         |         |          ]
Dd636         TGATCAATTTAATATTGTGTGATTATTTGGTGTGACGAACTCGGCAGTAATGATGGGCCTTACAGTTATTATTGTTTTATTGTTTTTAAATGGGGTGGAT
Dd432         ....................................................................................................
Lp_KC875348   ....................................................................................................
Lp_KC875349   ....................................................................................................
Lp_FR821799   ....................................................................................................
ChangesNS     ....................................................................................................

[             1801      1811      1821      1831      1841      1851      1861      1871      1881      1891       ]
[             |         |         |         |         |         |         |         |         |         |          ]
Dd636         CTCATCCCAAAAAGATGGCAATCTATTTTAGAGTTAACATATAGTCATTTTTATCGTGTTATAGAAGACAATYTGGGAGGGGAGGGGTTGAAGTATTTCT
Dd432         ........................................................................T...........................
Lp_KC875348   ........................................................................T...........................
Lp_KC875349   ........................................................................T...........................
Lp_FR821799   ........................................................................T...........................
ChangesNS     ........................................................................S...........................

[             1901      1911      1921      1931      1941      1951      1961      1971      1981      1991       ]
[             |         |         |         |         |         |         |         |         |         |          ]
Dd636         CTTTTGTTCTCTCTCTTTTTTTCTTTGGGGTTTGTTTGAATGTGCTGGGTTTATGCCCATATGTTTTTACTCCAACCGTTCATATTATAGTTACATTGGG
Dd432         .............................N......................................................................
Lp_KC875348   ....................................................................................................
Lp_KC875349   ....................................................................................................
Lp_FR821799   ....................................................................................................
ChangesNS     .............................S......................................................................
```

```
[            2001      2011      2021      2031      2041      2051      2061      2071      2081      2091      ]
[            |         |         |         |         |         |         |         |         |         |         ]
Dd636        TTTATCTTTTTCAATAATCATCGGTGTCACTCTTGCTGGTTTTTGGAGGTTTAAGTGAGATTTTTTTAGTGTTTTTATGCCAAGCGGAGCCCCTCTTGGG
Dd432        ....................................................................................................
Lp_KC875348  ....................................................................................................
Lp_KC875349  ....................................................................................................
Lp_FR821799  ....................................................................................................
ChangesNS    ....................................................................................................

[            2101      2111      2121      2131      2141      2151      2161      2171      2181      2191      ]
[            |         |         |         |         |         |         |         |         |         |         ]
Dd636        CTTGCCCCTTTGTTAGTTTTAATTGAAACAGTTAGTTATATCTCACGGGCTATTTCGTTAGGAGTTCGTTTGGCGGCTAATTTATCGGCTGGGCATCTAT
Dd432        ....................................................................................................
Lp_KC875348  ....................................................................................................
Lp_KC875349  ....................................................................................................
Lp_FR821799  ....................................................................................................
ChangesNS    ....................................................................................................

[            2201      2211      2221      2231      2241      2251      2261      2271      2281      2291      ]
[            |         |         |         |         |         |         |         |         |         |         ]
Dd636        TATTTGCTATTTTAGCTGGGTTTGGGTTTAATATGTTAATTGCAAGTGGGCCGGCGGGGCTATTGCCCTTGTTAATAATGGTTTTTATAACACTATTAGA
Dd432        ....................................................................................................
Lp_KC875348  .......G............................................................................................
Lp_KC875349  .......G............................................................................................
Lp_FR821799  .......G............................................................................................
ChangesNS    .......S............................................................................................

[            2301      2311      2321      2331      2341      2351      2361      2371      2381      2391      ]
[            |         |         |         |         |         |         |         |         |         |         ]
Dd636        AGTGGCGGTGGCCGTAATTCAGGCTTATGTTTTTTGTTTATTAACAACTATTTATTTAGCAGACACACTGATTTTACATATGGGTCTTTTTTGGCTAGTT
Dd432        ....................................................................................................
Lp_KC875348  ....................................................................................................
Lp_KC875349  ....................................................................................................
Lp_FR821799  ....................................................................................................
ChangesNS    ....................................................................................................

[            2401      2411      2421      2431      2441      2451      2461      2471      2481      2491      ]
[            |         |         |         |         |         |         |         |         |         |         ]
Dd636        CTTTTTGTTGGGATCATAAGTGTGACGGGGGCTTCAAGAGAAAAAAGGAGTGTGTTAAAAAAACGGGCCTTAGAGTGATCTATGGCTATTTTTTTTAGCT
Dd432        ....................................................................................................
Lp_KC875348  ....................................................................................................
Lp_KC875349  ....................................................................................................
Lp_FR821799  ....................................................................................................
ChangesNS    ....................................................................................................
```

```
[              2501      2511      2521      2531      2541      2551      2561      2571      2581      2591      ]
[              |         |         |         |         |         |         |         |         |         |         ]
Dd636          CTTTAGTTTTGTGGGGCGGATTTGACTGAGAAGGACATTTTCAATTTATTCATCAAATAGAATGAGAAATGCTTTTTATCTTGGACTGAGGCCCTATTAT
Dd432          ....................................................................................................
Lp_KC875348    ....................................................................................................
Lp_KC875349    ....................................................................................................
Lp_FR821799    ....................................................................................................
ChangesNS      ....................................................................................................

[              2601      2611      2621      2631      2641      2651      2661      2671      2681      2691      ]
[              |         |         |         |         |         |         |         |         |         |         ]
Dd636          TTTTGCCTTGGATGGTGTTTCCTTGTTTTTTTTGCTTTTAACAACTTTTTTAATACCGATTTGTGTTTTAATCAGTCAAAAATCTATCCGGTTTTTATTT
Dd432          ....................................................................................................
Lp_KC875348    ....................T...............................................................................
Lp_KC875349    ....................T...............................................................................
Lp_FR821799    ....................T...............................................................................
ChangesNS      ....................S...............................................................................

[              2701      2711      2721      2731      2741      2751      2761      2771      2781      2791      ]
[              |         |         |         |         |         |         |         |         |         |         ]
Dd636          AAAGAATTTCTTTTATGTTTATTTTTTTTAGAAGTGTTTTTAGTCGGTGTGTTTTTGGTGTTTGATCTTCTTTTATTTTATATTTTTTTTGAGGGGATTT
Dd432          ....................................................................................................
Lp_KC875348    ....................................................................................................
Lp_KC875349    ....................................................................................................
Lp_FR821799    ....................................................................................................
ChangesNS      ....................................................................................................

[              2801      2811      2821      2831      2841      2851      2861      2871      2881      2891      ]
[              |         |         |         |         |         |         |         |         |         |         ]
Dd636          TAATCCCAATGTTCTTTTTAATTGGAATTTGGGGGTCCCGAGAAGAAAAGGTTCGCGCTTCTTTTTATTTTTTTTTTTTTCNNACTTTTGCGGGGTCCGT
Dd432          ................................................................................---.................
Lp_KC875348    ................................................................................---.................
Lp_KC875349    ................................................................................---.................
Lp_FR821799    ................................................................................---.................
ChangesNS      ................................................................................---.................

[              2901      2911      2921      2931      2941      2951      2961      2971      2981      2991      ]
[              |         |         |         |         |         |         |         |         |         |         ]
Dd636          GTTTTTTTTTTTTACAATCCTTTTTTTGTATCGAACAACAGGGGCAACAGATTATTTTCTTTTGCTTAATCTTAGGCTGTCTCCCAATGTTCAGAAGTGG
Dd432          ....................................................................................................
Lp_KC875348    ....................................................................................................
Lp_KC875349    ....................................................................................................
Lp_FR821799    ....................................................................................................
ChangesNS      ....................................................................................................
```

```
[             3001      3011      3021      3031      3041      3051      3061      3071      3081      3091       ]
[             |         |         |         |         |         |         |         |         |         |          ]
Dd636         GCCTTAATTGGCGTCTTTCTTAGTTTTGCAGTTAAAATTCCCTTAATCCCGTTTCATATTTGGCTTCCCCAAGCACATGTGGAAGCCCCTGTTGCGGGCT
Dd432         ....................................................................................................
Lp_KC875348   ....................................................................................................
Lp_KC875349   ....................................................................................................
Lp_FR821799   ....................................................................................................
ChangesNS     ....................................................................................................

[             3101      3111      3121      3131      3141      3151      3161      3171      3181      3191       ]
[             |         |         |         |         |         |         |         |         |         |          ]
Dd636         CAGTTATTTTAGCTGGGATTTTATTAAAACTAGGGGGGTATGGGGTTTTACGGTTTTCTTGGCCTCTCTTCCCTGCGGCTTCTGAATATTGATCTCCTGT
Dd432         .........................................................................C..........................
Lp_KC875348   ....................................................................................................
Lp_KC875349   ....................................................................................................
Lp_FR821799   ....................................................................................................
ChangesNS     ....................................................................................................

[             3201      3211      3221      3231      3241      3251      3261      3271      3281      3291       ]
[             |         |         |         |         |         |         |         |         |         |          ]
Dd636         TATTGTTTTTTTTTCTGWTTTGGCYGTTGTTTATGGGGGTTTAATGACATGTCGTCAGGTTGACTTTAAACGACTAGTTGCTTATTCTTCGGTGGCACAC
Dd432         .................T......T...........................................................................
Lp_KC875348   .................T......T...........................................................................
Lp_KC875349   .................T......T...........................................................................
Lp_FR821799   .................T......T...........................................................................
ChangesNS     .................S......S...........................................................................

[             3301      3311      3321      3331      3341      3351      3361      3371      3381      3391       ]
[             |         |         |         |         |         |         |         |         |         |          ]
Dd636         ATGGGTTTAGTTCCTCTAGGGGTTTTTACACATATTATAGAAGGGCTTGTTGGAGCTGTTTTTTTAATGTTAGCCCATGGTTTTGTTAGTTCCGCTCTTT
Dd432         .....CC.............................................................................................
Lp_KC875348   .....C..............................................................................................
Lp_KC875349   .....C..............................................................................................
Lp_FR821799   .....C..............................................................................................
ChangesNS     .....SS.............................................................................................

[             3401      3411      3421      3431      3441      3451      3461      3471      3481      3491       ]
[             |         |         |         |         |         |         |         |         |         |          ]
Dd636         TTATTGGGATTACGTATTTATATGATCGCCATCATACTCGTTTAATTAAATATTATCGTGGCTTGGCTTTAACGATGCCGCTTTTTGCTATTTCAATGTT
Dd432         ....................................................................................................
Lp_KC875348   ....................................................................................................
Lp_KC875349   ....................................................................................................
Lp_FR821799   ....................................................................................................
ChangesNS     ....................................................................................................
```

```
[           3501      3511      3521      3531      3541      3551      3561      3571      3581      3591      ]
[           |         |         |         |         |         |         |         |         |         |         ]
Dd636       AATTTTGTCCTTAACAAACATGGGCTTCCCGTTAAGTAGTAATTTTGTTGGAGAGTTTTTTTCTTTGTTAGCAGCTTTTAAGTATCATTTGGGGGTTGGG
Dd432       ...............................................................Y....................................
Lp_KC875348 ....................................................................................................
Lp_KC875349 ....................................................................................................
Lp_FR821799 ....................................................................................................
ChangesNS   ...............................................................S....................................

[           3601      3611      3621      3631      3641      3651      3661      3671      3681      3691      ]
[           |         |         |         |         |         |         |         |         |         |         ]
Dd636       GGTTTTGTTGTTTTAGGAGTTATTTTTTCTGTTGTTTATTCTCTTAGTTTGTTTAWTCGGATTTCTTTTGGGGGYGGTTCTAATTATCTTCTTTTTAACA
Dd432       .......................................................A..................C.........................
Lp_KC875348 .......................................................A..................C.........................
Lp_KC875349 .......................................................A..................C.........................
Lp_FR821799 .......................................................A..................C.........................
ChangesNS   .......................................................S..................S.........................

[           3701      3711      3721      3731      3741      3751      3761      3771      3781      3791      ]
[           |         |         |         |         |         |         |         |         |         |         ]
Dd636       GAGATTTAAGTCGACAAGAAGCTTTTGTCATGCTTCCTTTTCTTGTAATTATTTTTTTTGGGGGCGTTGTCCCTTTTTTTATTCTTGATTTAATAAGAAA
Dd432       ....................................................................................................
Lp_KC875348 ....................................................................................................
Lp_KC875349 ....................................................................................................
Lp_FR821799 ....................................................................................................
ChangesNS   ....................................................................................................

[           3801      3811      3821      3831      3841      3851      3861      3871      3881      3891      ]
[           |         |         |         |         |         |         |         |         |         |         ]
Dd636       TTGTCTTGTGTTTAGTCCGATTGGATATGACTATCATCTTGTGGAGTTTTCTCCTTGACCTTTTATTGGAGCTGCCGGGGCCTTCTTCTTGACTGTGGGG
Dd432       ....................................................................................................
Lp_KC875348 ....................................................................................................
Lp_KC875349 ....................................................................................................
Lp_FR821799 ....................................................................................................
ChangesNS   ....................................................................................................

[           3901      3911      3921      3931      3941      3951      3961      3971      3981      3991      ]
[           |         |         |         |         |         |         |         |         |         |         ]
Dd636       GCAGTTGTTTTTTTTCATTATGGTTTGACTTTTTTTTTGGGTTTAGGGGCGCTGATTGTACTTGGGGTGATGTTTGTTTGATGACAAGACATTATACGAG
Dd432       ....................................................................................................
Lp_KC875348 ....................................................................................................
Lp_KC875349 ....................................................................................................
Lp_FR821799 ....................................................................................................
ChangesNS   ....................................................................................................
```

```
[              4001      4011      4021      4031      4041      4051      4061      4071      4081      4091       ]
[              |         |         |         |         |         |         |         |         |         |          ]
Dd636          AAGCGACTTTTCAGGGACACCATTCTTTAGTTGTAAAGCAAGGCCTTAAGTATGGCATGCTTTTGTTTATTCTTTCAGAAGTTTGTTTTTTTTTTTCTTT
Dd432          ....................................................................................................
Lp_KC875348    ....................................................................................................
Lp_KC875349    ....................................................................................................
Lp_FR821799    ....................................................................................................
ChangesNS      ....................................................................................................

[              4101      4111      4121      4131      4141      4151      4161      4171      4181      4191       ]
[              |         |         |         |         |         |         |         |         |         |          ]
Dd636          TTTTTGAGCTTTTTTTCATAGTAGTTTGGCTCCGGCTATTGAACTTGGGGTTGTGTGACCACCGCAAGGTGTTCATGCGCTAAATCCTTTTTCTGTTCCA
Dd432          ....................................................................................................
Lp_KC875348    ....................................................................................................
Lp_KC875349    ....................................................................................................
Lp_FR821799    ....................................................................................................
ChangesNS      ....................................................................................................

[              4201      4211      4221      4231      4241      4251      4261      4271      4281      4291       ]
[              |         |         |         |         |         |         |         |         |         |          ]
Dd636          TTGTTAAGCACGGCCGTTTTATTGAGTTCTGGGGCATCGGTAACGTGGGCCCATCATGCTATAATAAGTGGGAATAAGAAAGAAGCGGTTGCAGGTTTGT
Dd432          .......A................G...........................................................................
Lp_KC875348    .......A............................................................................................
Lp_KC875349    .......A............................................................................................
Lp_FR821799    .......A............................................................................................
ChangesNS      .......N................N...........................................................................

[              4301      4311      4321      4331      4341      4351      4361      4371      4381      4391       ]
[              |         |         |         |         |         |         |         |         |         |          ]
Dd636          CTTTGACTATTTTATTGGGTGTGGTGTTTACAGGTTTACAGGCCCTTGAATATTATGAAGCTCCCTTTGCTCTTTCCGATTCTGTTTATGGCTCCACTTT
Dd432          ...............................................................................C....................
Lp_KC875348    ....................................................................................................
Lp_KC875349    ....................................................................................................
Lp_FR821799    ....................................................................................................
ChangesNS      ...............................................................................S....................

[              4401      4411      4421      4431      4441      4451      4461      4471      4481      4491       ]
[              |         |         |         |         |         |         |         |         |         |          ]
Dd636          TTTTGTTGCAACAGGGTTTCATGGGTTACACGTGATAATTGGAACAACTTTTTTGTTTGTTTGTTTTCTTCGGTTACTTTCCAATCAGTTTACCCGTAGC
Dd432          ....................................................................................................
Lp_KC875348    .................................................................................................G..
Lp_KC875349    .................................................................................................G..
Lp_FR821799    .................................................................................................G..
ChangesNS      .................................................................................................N..
```

```
[            4501      4511      4521      4531      4541      4551      4561      4571      4581      4591       ]
[            |         |         |         |         |         |         |         |         |         |          ]
Dd636        CAACATCTGGGTTTTGAAGCGGCAAGTTGATATTGGCATTTTGTCGATGTTGTTTGGTTGTTTTTATATCTTTCAATTTATTTGTGGGGTTCTAGAGATG
Dd432        ....................................................................................................
Lp_KC875348  .......................C............................................................................
Lp_KC875349  .......................C............................................................................
Lp_FR821799  .......................C............................................................................
ChangesNS    .......................S............................................................................

[            4601      4611      4621      4631      4641      4651      4661      4671      4681      4691       ]
[            |         |         |         |         |         |         |         |         |         |          ]
Dd636        AGCCAGAGGCTTGGGTTTTGGGGTTTCAGGATGTGGCAGACCCGGTAGTAGAAGAAATTGTTTTTTTTCATGATCAAGTAATGTTTTTATTAATCATTAT
Dd432        ....................................................................................................
Lp_KC875348  ....................................................................................................
Lp_KC875349  ....................................................................................................
Lp_FR821799  ....................................................................................................
ChangesNS    ....................................................................................................

[            4701      4711      4721      4731      4741      4751      4761      4771      4781      4791       ]
[            |         |         |         |         |         |         |         |         |         |          ]
Dd636        TGTCACTGTTGTTTTATGGCTTATTGTGGAAGCTTTTTGAAATAAATTTTATGATCGTAATTTAGTTGATGGTACTTTTTTAGAAATTGTTTGAACAATA
Dd432        ....................................................................................................
Lp_KC875348  ....................................................................................................
Lp_KC875349  ....................................................................................................
Lp_FR821799  ....................................................................................................
ChangesNS    ....................................................................................................

[            4801      4811      4821      4831      4841      4851      4861      4871      4881      4891       ]
[            |         |         |         |         |         |         |         |         |         |          ]
Dd636        ATCCCCGCTGTTATATTGATTTTTATTGCACTACCCTCGTTAAAATTGTTGTATTTAATGGACGAAATCATTTCTCCTGCTTTAACAATTAAAGTCATTG
Dd432        ....................................................................................................
Lp_KC875348  ....................................................................................................
Lp_KC875349  ....................................................................................................
Lp_FR821799  ....................................................................................................
ChangesNS    ....................................................................................................

[            4901      4911      4921      4931      4941      4951      4961      4971      4981      4991       ]
[            |         |         |         |         |         |         |         |         |         |          ]
Dd636        GCCATCAATGATATTGGTCTTATGAATACTCTGATTATGAAGGCGAGACGTTAGCTTTTGATTCTTATATGGTTCCGTCTTCGGATTTAATTTCAGGGAA
Dd432        ....................................................................................................
Lp_KC875348  ....................................................................................................
Lp_KC875349  ....................................................................................................
Lp_FR821799  ....................................................................................................
ChangesNS    ....................................................................................................
```

```
[             5001      5011      5021      5031      5041      5051      5061      5071      5081      5091      ]
[             |         |         |         |         |         |         |         |         |         |         ]
Dd636         AAACCGTTTACTTGAAGTGGATCAAAAACTTGTTGTTCCAATTGGAACTCATATAAGATTTTTAGTGACGGGAGCCGATGTCTTGCATTCTTTTGCGGTC
Dd432         .......................................................G............................................
Lp_KC875348   ....................................................................................................
Lp_KC875349   ....................................................................................................
Lp_FR821799   ....................................................................................................
ChangesNS     .......................................................N............................................

[             5101      5111      5121      5131      5141      5151      5161      5171      5181      5191      ]
[             |         |         |         |         |         |         |         |         |         |         ]
Dd636         CCTTCTTTAGGATTAAAAGTAGACGCTGTGCCTGGCCGTTTAAATCAAACTGGTGTTTTTATCAAACGACCGGGGGTTTTTTTT---GGGCAATGCTCTG
Dd432         ....................................................................................TNN.............
Lp_KC875348   ....................................................................................---.............
Lp_KC875349   ....................................................................................---.............
Lp_FR821799   ....................................................................................---.............
ChangesNS     ....................................................................................---.............

[             5201      5211      5221      5231      5241      5251      5261      5271      5281      5291      ]
[             |         |         |         |         |         |         |         |         |         |         ]
Dd636         AGATTTGTGGGGCGAATCACTCTTTTATGCCTATTGTTATAGAGGGAGTCGGGTTAAATGAATATATTATGTATTATAAGTATTTAGTTGTTGTCATGGT
Dd432         ....................................................................................................
Lp_KC875348   ....................................................................................................
Lp_KC875349   ....................................................................................................
Lp_FR821799   .................................................................---................................
ChangesNS     .................................................................---................................

[             5301      5311      5321      5331      5341      5351      5361      5371      5381      5391      ]
[             |         |         |         |         |         |         |         |         |         |         ]
Dd636         TTTATTTTTATTAGGAGGTTGGGGGGTAGTTTTAAATCGAGGACATTTTATTATAATGATTGTTTCAATTGAACTTGTTTTATTAGCAACTTTTTTTTTT
Dd432         ....................................................................................................
Lp_KC875348   ....................................................................................................
Lp_KC875349   ....................................................................................................
Lp_FR821799   ....................................................................................................
ChangesNS     ....................................................................................................

[             5401      5411      5421      5431      5441      5451      5461      5471      5481      5491      ]
[             |         |         |         |         |         |         |         |         |         |         ]
Dd636         TTTTTTNNGATAAATTCTAAGGAAATAGACGCTTTAATAGAACAGGTTTTTATGATAATGGGTTTGACAATTGCGGCGGCAGAGTCTTCTATTGGCTTGG
Dd432         ....N---.........................Y..................................................................
Lp_KC875348   .....---............................................................................................
Lp_KC875349   .....---............................................................................................
Lp_FR821799   .....---............................................................................................
ChangesNS     ....S---.........................S..................................................................
```

```
[           5501      5511      5521      5531      5541      5551      5561      5571      5581      5591      ]
[           |         |         |         |         |         |         |         |         |         |         ]
Dd636       CTCTTTTAGTGGCCTATTACCGAATTAGGGGAACGATTGTTTTAAAATCCTTTAGTTCTTTACGGGTTTTTTTTTTTGTTTTAATAGCAGTTGTCTTAGC
Dd432       ....................................................................................................
Lp_KC875348 ....................................................................................................
Lp_KC875349 ....................................................................................................
Lp_FR821799 ....................................................................................................
ChangesNS   ....................................................................................................

[           5601      5611      5621      5631      5641      5651      5661      5671      5681      5691      ]
[           |         |         |         |         |         |         |         |         |         |         ]
Dd636       CGGGGTATTTTCTAGTATTTCTTTTTTTATTGGAGAAAAAAGACCGGACCGAGAAAAAGTGTCTGCTTATGAGTGTGGGTTTGTACCTTTTAGTTTTCTG
Dd432       ....................................................................................................
Lp_KC875348 ....................................................................................................
Lp_KC875349 ....................................................................................................
Lp_FR821799 ....................................................................................................
ChangesNS   ....................................................................................................

[           5701      5711      5721      5731      5741      5751      5761      5771      5781      5791      ]
[           |         |         |         |         |         |         |         |         |         |         ]
Dd636       GGGCGCCCCTTTTCTGTGCGGTTCTTTTTAATTGGTATTTTGTTCTTAATTTTTGACTTAGAAATTTCTTTTTTATTTCCTTGGTGTGTTTTATATAATT
Dd432       ....................................................................................................
Lp_KC875348 ....................................................................................................
Lp_KC875349 ....................................................................................................
Lp_FR821799 ....................................................................................................
ChangesNS   ....................................................................................................

[           5801      5811      5821      5831      5841      5851      5861      5871      5881      5891      ]
[           |         |         |         |         |         |         |         |         |         |         ]
Dd636       CACTTGGACCTTTTGGGTTTTGAGCCATGGTTGGGTTTTTGTTTATATTAACTGTTGGCCTGGTTTATGAGTGACTAAAAGGAGGGCTAGAATGGGAGCC
Dd432       ....................................................................................................
Lp_KC875348 ....................................................................................................
Lp_KC875349 ....................................................................................................
Lp_FR821799 ....................................................................................................
ChangesNS   ....................................................................................................

[           5901      5911      5921      5931      5941      5951      5961      5971      5981      5991      ]
[           |         |         |         |         |         |         |         |         |         |         ]
Dd636       AACTCCGGTTTCTGCCTTAATCCATGCGGCAACAATGGTTACGGCAGGTGTTTTTTTGTTAATTAGAGCTTCCCCTCTTTTTGATGTYGTTCCTTTTATG
Dd432       ....................................................................................................
Lp_KC875348 .......................................................................................C............
Lp_KC875349 .......................................................................................C............
Lp_FR821799 .......................................................................................C............
ChangesNS   .......................................................................................S............
```

```
[            6001      6011      6021      6031      6041      6051      6061      6071      6081      6091      ]
[            |         |         |         |         |         |         |         |         |         |         ]
Dd636        TTGGTTTTTATAACGATAATAGGGGTGTTAACAGTTTTTGTTGCGGGAACAATTGGTCTTGTTCAAAATGATTTAAAAAAAATAATTGCTTATTCCACTT
Dd432        ....................................................................................................
Lp_KC875348  ....................................................................................................
Lp_KC875349  ....................................................................................................
Lp_FR821799  ....................................................................................................
ChangesNS    ....................................................................................................

[            6101      6111      6121      6131      6141      6151      6161      6171      6181      6191      ]
[            |         |         |         |         |         |         |         |         |         |         ]
Dd636        GTAGTCAATTGGGGTATATGGTTGTGGCTTGTGGTCTTTCTCATTTTTCTATTGGTCTTTTCCACTTAATGAATCATGCTTTTTTTAAGGCTTTGTTATT
Dd432        ....................................................................................................
Lp_KC875348  ....................................................................................................
Lp_KC875349  ....................................................................................................
Lp_FR821799  ....................................................................................................
ChangesNS    ....................................................................................................

[            6201      6211      6221      6231      6241      6251      6261      6271      6281      6291      ]
[            |         |         |         |         |         |         |         |         |         |         ]
Dd636        TTTAAGTGCTGGTTCTTTAATTCATGCAATGATAGACGAACAAGACATAAGAAAAATGGGGGGCTTATTACAAATCAYACCTTTGACTTATATTTTTTTT
Dd432        .............................................................................T......................
Lp_KC875348  .............................................................................T......................
Lp_KC875349  .............................................................................T......................
Lp_FR821799  .............................................................................T......................
ChangesNS    .............................................................................S......................

[            6301      6311      6321      6331      6341      6351      6361      6371      6381      6391      ]
[            |         |         |         |         |         |         |         |         |         |         ]
Dd636        ATTATAGGCTCTTTTTCTTTAATGGGATTTCCTTTTTTAACCGGGTTTTATTCAAAAGACTTAATCTTAGRAGTTACTTTTGGGCAATATTATTTAATTT
Dd432        ......................................................................A.............................
Lp_KC875348  ......................................................................A.............................
Lp_KC875349  ......................................................................A.............................
Lp_FR821799  ......................................................................A.............................
ChangesNS    ......................................................................S.............................

[            6401      6411      6421      6431      6441      6451      6461      6471      6481      6491      ]
[            |         |         |         |         |         |         |         |         |         |         ]
Dd636        TTGTTTATTGGCTAGGTTGTTTTTCTGTTTTATTAACAGCAATGTATTCAATCCGTTTGGTTYATTATGCCTTTTTCTCTAATACAAATTCTAAAAGGGC
Dd432        ..............................................................T.....................................
Lp_KC875348  ..............................................................T.....................................
Lp_KC875349  ..............................................................T.....................................
Lp_FR821799  ..............................................................T.....................................
ChangesNS    ..............................................................S.....................................
```

```
[               6501      6511      6521      6531      6541      6551      6561      6571      6581      6591       ]
[               |         |         |         |         |         |         |         |         |         |          ]
Dd636           GGTTTTTGCCTCGTTAAAAGAGGGAGAGGGCCTTTTGTTGGCCCCTTTGGGGGTTTTAGCCTTAGGTAGTCTTTTTTGGGGTTATTTATGTAAAGAAATT
Dd432           ....................................................................................................
Lp_KC875348     ....................................................................................................
Lp_KC875349     ....................................................................................................
Lp_FR821799     ....................................................................................................
ChangesNS       ....................................................................................................

[               6601      6611      6621      6631      6641      6651      6661      6671      6681      6691       ]
[               |         |         |         |         |         |         |         |         |         |          ]
Dd636           GTGTGGTCTTTTCAAATGGGGCTTTCGCCAATGCTTTTTTTTAAAATAAAAATGCTTCCTGTTATAGTAAGTATTATGGGGGCATTGGGGGTTATTTTTG
Dd432           ....................................................................................................
Lp_KC875348     ....................................................................................................
Lp_KC875349     ....................................................................................................
Lp_FR821799     ....................................................................................................
ChangesNS       ....................................................................................................

[               6701      6711      6721      6731      6741      6751      6761      6771      6781      6791       ]
[               |         |         |         |         |         |         |         |         |         |          ]
Dd636           TTTTATTTAATTTTTCTTCTAAAATATTGTTTTTTTTGTTTCTCTTTATACCTTTTTTTGGCTCTGCTTGACAAATAAATTCTTTTTTTAATTTTTTTTT
Dd432           ...................C................................................................................
Lp_KC875348     ....................................................................................................
Lp_KC875349     ....................................................................................................
Lp_FR821799     ....................................................................................................
ChangesNS       ...................S................................................................................

[               6801      6811      6821      6831      6841      6851      6861      6871      6881      6891       ]
[               |         |         |         |         |         |         |         |         |         |          ]
Dd636           TATAAAAAAAATCTATAAAACGGGGCATCTGATTATGAATTTAACTGTTGACAAAGGTGTCTTAGAGCTGGTTGGGCCCAGGGGCCTTGTTCAATTTCTA
Dd432           ....................................................................................................
Lp_KC875348     ....................................................................................................
Lp_KC875349     ....................................................................................................
Lp_FR821799     ................................................................A...................................
ChangesNS       ................................................................N...................................

[               6901      6911      6921      6931      6941      6951      6961      6971      6981      6991       ]
[               |         |         |         |         |         |         |         |         |         |          ]
Dd636           GTCCGCCAAATTCAAAGACTTAGTGATTTACAGTCGGGGCAAGTTTTTAATTATGCTTTAGTTATACTAATTGGTGTTGTCATATTTATTTGGGGAAAAG
Dd432           ....................................................................................................
Lp_KC875348     ....................................................................................................
Lp_KC875349     ....................................................................................................
Lp_FR821799     ....................................................................................................
ChangesNS       ....................................................................................................
```

```
[           7001      7011      7021      7031      7041      7051      7061      7071      7081      7091      ]
[           |         |         |         |         |         |         |         |         |         |         ]
Dd636       AAAACCCTCTTTTGTCTCTGGTCAATGGATTCTTAGTGAATTTGCCCTCTCCCTCCAACATAAGTTATTTTTGAAATTTTGGGTCTTTATTGGGCCTGTG
Dd432       ....................................................................................................
Lp_KC875348 ....................................................................................................
Lp_KC875349 ....................................................................................................
Lp_FR821799 ....................................................................................................
ChangesNS   ....................................................................................................

[           7101      7111      7121      7131      7141      7151      7161      7171      7181      7191      ]
[           |         |         |         |         |         |         |         |         |         |         ]
Dd636       TTTGGTGCTACAAATTATAACAGGCTGTTTTTTGTCTATGCATTATTGTCCAGATGTTAATTTAGCTTTTGCTTCTATCGGCCATATTATGCGGGATGTG
Dd432       ....................................................................................................
Lp_KC875348 ....................................................................................................
Lp_KC875349 ....................................................................................................
Lp_FR821799 ....................................................................................................
ChangesNS   ....................................................................................................

[           7201      7211      7221      7231      7241      7251      7261      7271      7281      7291      ]
[           |         |         |         |         |         |         |         |         |         |         ]
Dd636       AACTCTGGTTTTTTATTAAAATATTTACATGCAAATGGTGCTGCTTTATTTTTTTTTTGTCTTTATGTTCATGTTGGGCGGGGCTTGTATTATGGGAGTT
Dd432       .............................................................................T......................
Lp_KC875348 ....................................................................................................
Lp_KC875349 ....................................................................................................
Lp_FR821799 ....................................................................................................
ChangesNS   .............................................................................S......................

[           7301      7311      7321      7331      7341      7351      7361      7371      7381      7391      ]
[           |         |         |         |         |         |         |         |         |         |         ]
Dd636       ATTTGAAATTTCATGTTTGAAGTGTTGGGGTTGTAATTTTTTTATTAACGATGGCGACGGCTTTTATGGGTTATGTTTTGCCTTGAGGGCAAATGTCTTT
Dd432       ....................................................................................................
Lp_KC875348 ....................................................................................................
Lp_KC875349 ....................................................................................................
Lp_FR821799 ....................................................................................................
ChangesNS   ....................................................................................................

[           7401      7411      7421      7431      7441      7451      7461      7471      7481      7491      ]
[           |         |         |         |         |         |         |         |         |         |         ]
Dd636       TTGAGGAGCCACTGTTATCACAAATTTATTGTCTGCTATTCCTTACTTTGGGGTAGACATTGTTCAATGGGTTTGAGGGGGTTTTAGTGTTTCTGGGGCA
Dd432       ....................................................................................................
Lp_KC875348 ....................................................................................................
Lp_KC875349 ....................................................................................................
Lp_FR821799 ..........C.........................................................................................
ChangesNS   ..........N.........................................................................................
```

```
[           7501      7511      7521      7531      7541      7551      7561      7571      7581      7591       ]
[           |         |         |         |         |         |         |         |         |         |          ]
Dd636       ACCTTAAATCGGTTTTTTAGTTTACATTTTTTACTTCCTTTTTTTTTGGTTGTTTTTGTTTTTATTCATTTAATGTATTTACATGTTGATGGGTCAAATA
Dd432       ....................................................................................................
Lp_KC875348 ....................................................................................................
Lp_KC875349 ....................................................................................................
Lp_FR821799 ....................................................................................................
ChangesNS   ....................................................................................................

[           7601      7611      7621      7631      7641      7651      7661      7671      7681      7691       ]
[           |         |         |         |         |         |         |         |         |         |          ]
Dd636       ACCCGACGGGGTTRAACTCTTCTAATGAGAATGTTTCTTTCCATACTTTTTATACTTCAAAAGATCTTTTTGGGTTTTTTTTTCTTTTTATTTATTTTTG
Dd432       .............A......................................................................................
Lp_KC875348 .............A......................................................................................
Lp_KC875349 .............A......................................................................................
Lp_FR821799 .............A......................................................................................
ChangesNS   .............S......................................................................................

[           7701      7711      7721      7731      7741      7751      7761      7771      7781      7791       ]
[           |         |         |         |         |         |         |         |         |         |          ]
Dd636       TTTGTTTGTTTTTTTTTGACCTAATTTGTTGGGGGACTCGGAAARCTTTATTCAAGCGAACTCTTTGGTCACTCCTGTGCACATTCAGCCAGAATGGTAT
Dd432       ............................................A....R..................................................
Lp_KC875348 ............................................A.......................................................
Lp_KC875349 ............................................A.......................................................
Lp_FR821799 ............................................A.......................................................
ChangesNS   ............................................S....S..................................................

[           7801      7811      7821      7831      7841      7851      7861      7871      7881      7891       ]
[           |         |         |         |         |         |         |         |         |         |          ]
Dd636       TTTTTATTTGCTTATGCAATCTTGCGTTCAATACCCAATAAGTTGGGGGGGGTCATTGCAATGTTTTGTAGCATTTTAGTTTTGTTTTTACTACCAATTT
Dd432       .......................................................................T............................
Lp_KC875348 ....................................................................................................
Lp_KC875349 ....................................................................................................
Lp_FR821799 ....................................................................................................
ChangesNS   .......................................................................S............................

[           7901      7911      7921      7931      7941      7951      7961      7971      7981      7991       ]
[           |         |         |         |         |         |         |         |         |         |          ]
Dd636       TACACAAAAGTGTACTAAAGGGGTGTTCTTTTCGCCCTCTTGGGCGTGTCGCCTTTTGATTTTTGCTTGTTGATTTCGGCCTTTTAACTTGAATTGGGGC
Dd432       ........................................................................................Y...........
Lp_KC875348 ....................................................................................................
Lp_KC875349 ....................................................................................................
Lp_FR821799 ....................................................................................................
ChangesNS   ........................................................................................S...........
```

```
[              8001      8011      8021      8031      8041      8051      8061      8071      8081      8091       ]
[              |         |         |         |         |         |         |         |         |         |          ]
Dd636          ACAGGTAGTCGAAGAGCCTTTTATTACAATTGGTCAGATTCTTTCTTTTTTTTATTTTTTTTATTTTTTAGTTCTTGTTCCTGTCCTTGGTCTTTTAGAA
Dd432          ........Y.....................................................................................C.G...
Lp_KC875348    ........................A.......................................................................T...
Lp_KC875349    ........................A.......................................................................T...
Lp_FR821799    ....................................................................................................
ChangesNS      ........S...............S.....................................................................N.N...

[              8101      8111      8121      8131      8141      8151      8161      8171      8181      8191       ]
[              |         |         |         |         |         |         |         |         |         |          ]
Dd636          AACCAATTATTAATTATTGTTTTTGGTATTTTTTGTTTAATTTCTTCTACTAATTGACTTTCTGTTTATTTAGCAATCGAACTTTCTACTCTTTGTTTTT
Dd432          ....................................................................................................
Lp_KC875348    ....................................................................................................
Lp_KC875349    ....................................................................................................
Lp_FR821799    ....................................................................................................
ChangesNS      ....................................................................................................

[              8201      8211      8221      8231      8241      8251      8261      8271      8281      8291       ]
[              |         |         |         |         |         |         |         |         |         |          ]
Dd636          TTGTTTTAATTGCCCGCGGATCGGGGTATAGCGCAGAAGCAGGGTTAAAGTACTTTGTTTTAGGTGCGCTTTCTTCTGGTTTGTTTTTATTTGGGTGTGC
Dd432          ....................................................T...............................................
Lp_KC875348    ....................................................T...............................................
Lp_KC875349    ....................................................T...............................................
Lp_FR821799    ....................................................T...............................................
ChangesNS      ....................................................S...............................................

[              8301      8311      8321      8331      8341      8351      8361      8371      8381      8391       ]
[              |         |         |         |         |         |         |         |         |         |          ]
Dd636          TTTATTATGTGGTATTGGGGGGAATGTACATCTGGCATATCTAGATCTAATTATTAACTCGAAACAAACTTTTTCGGATGTCTGTCCTCCGGTCGGGTAT
Dd432          ..........................................................................................A.........
Lp_KC875348    ..........................................................................................A.........
Lp_KC875349    ..........................................................................................A.........
Lp_FR821799    ..........................................................................................A.........
ChangesNS      ..........................................................................................S.........

[              8401      8411      8421      8431      8441      8451      8461      8471      8481      8491       ]
[              |         |         |         |         |         |         |         |         |         |          ]
Dd636          ATTTTAATTTTAGGGGCCCTTTTTTTTAAATTGTCTGTTGCTCCTTTTCATATGTGGGCTCCAGATGTATATGAAGGAGCCCCAACAAAAATTGTTTTAT
Dd432          ....................................................................................................
Lp_KC875348    ....................................................................................................
Lp_KC875349    ....................................................................................................
Lp_FR821799    ....................................................................................................
ChangesNS      ....................................................................................................
```

```
[            8501      8511      8521      8531      8541      8551      8561      8571      8581      8591         ]
[            |         |         |         |         |         |         |         |         |         |            ]
Dd636        TATTGGCCACTGTGCCGAAGATAGGGATTTTYTCTCTTTTAATTGCGCTCGGTTTGCCAGTTAATTCTTTATTAATTGGGGTTGTTTTATCTTTATTTGT
Dd432        ...............................T..........................G........Y....................T...........
Lp_KC875348  ...............................T..........................G.............................T...........
Lp_KC875349  ...............................T..........................G.............................T...........
Lp_FR821799  ...............................T..........................G.............................T...........
ChangesNS    ...............................S..........................S.............................N...........

[            8601      8611      8621      8631      8641      8651      8661      8671      8681      8691         ]
[            |         |         |         |         |         |         |         |         |         |            ]
Dd636        TGGAACTTTAGGGGCCTTAAACCAAACAAAAATTAAACGACTATTGGCCTATAGTAGTATTGGTCATATGGGCTTTATTCTATGGGGCTTCGAGAGTGGT
Dd432        ......K.............................................................................................
Lp_KC875348  ....................................................................................................
Lp_KC875349  ....................................................................................................
Lp_FR821799  ....................................................................................................
ChangesNS    ......S.............................................................................................

[            8701      8711      8721      8731      8741      8751      8761      8771      8781      8791         ]
[            |         |         |         |         |         |         |         |         |         |            ]
Dd636        TCCTTTGAAAGTTTACAAGCCAGTTTGGTCYATCTTTTYATATATGTTATTATGACTATCTGTGTTTTTTCTCTTATATTGGGCTTTCGTTTATATAAGA
Dd432        ..............................T.......T.................................................A...........
Lp_KC875348  ..............................T.......T.................................................A...........
Lp_KC875349  ..............................T.......T.................................................A...........
Lp_FR821799  ..............................T.......T.................................................A...........
ChangesNS    ..............................S.......S.................................................N...........

[            8801      8811      8821      8831      8841      8851      8861      8871      8881      8891         ]
[            |         |         |         |         |         |         |         |         |         |            ]
Dd636        ATTTACTTATAGAATTTAGTGGGGTGTCTCGATTTTTACCTCTTTTTGCCGTTACTTTAGGCGTCGTATTTTTTTCTATTGCTGGAATTCCTCCTTTTGC
Dd432        ....................................................................................................
Lp_KC875348  ........................................................................................C...........
Lp_KC875349  ........................................................................................C...........
Lp_FR821799  ........................................................................................C...........
ChangesNS    ........................................................................................S...........

[            8901      8911      8921      8931      8941      8951      8961      8971      8981      8991         ]
[            |         |         |         |         |         |         |         |         |         |            ]
Dd636        AGGATTTTTAAGTAAATGAGTTGTTTTGTTGTCTGGAGTACTTTCTCAGTCTTATTTTGTTTTTCTATTTGCCGTTTTTTGTTCTGTAATAGGGGGTGTT
Dd432        .............................................................................................A......
Lp_KC875348  .............................................................................................A......
Lp_KC875349  .............................................................................................A......
Lp_FR821799  .............................................................................................A......
ChangesNS    .............................................................................................S......
```

```
[           9001      9011      9021      9031      9041      9051      9061      9071      9081      9091      ]
[           |         |         |         |         |         |         |         |         |         |         ]
Dd636       TATTATGTTAGAATTGTCAAAATACTTTTTTTTCAAAAAAACTCTTATCTTTTAATTACGATAAAGCTCTAAGAAAAGAATTACACTTAAATTTTAAAA
Dd432       ....................................................................................................
Lp_KC875348 ....................................................................................................
Lp_KC875349 ....................................................................................................
Lp_FR821799 ....................................................................................................
ChangesNS   ....................................................................................................

[           9101      9111      9121      9131      9141      9151      9161      9171      9181      9191      ]
[           |         |         |         |         |         |         |         |         |         |         ]
Dd636       AAGTCTTTTTGGTTGGTCTTTGTCTTTATTTATTCTGTTTCTTTTTGTGGCTCCTCATTTAGGTTTTTTTTTTGCTTCTCAAACAATAATGATATTGTT
Dd432       ....................................................................................................
Lp_KC875348 ....................................................................................................
Lp_KC875349 ....................................................................................................
Lp_FR821799 ............................................................C.......................................
ChangesNS   ............................................................N.......................................

[           9201      9211      9221      9231      9241      9251      9261      9271      9281      9291      ]
[           |         |         |         |         |         |         |         |         |         |         ]
Dd636       TTTTGGGGCAATTGGTTCTGGGATAATGGTTATTTCTGCATTAAACCCTGTATTTTCTCTTTTTTGATTGGTTATCGTTTTTATTAATTCTGCAGTTTTT
Dd432       ..............................Y.....................Y...............................................
Lp_KC875348 ....................................................................................................
Lp_KC875349 ....................................................................................................
Lp_FR821799 ...........................................................C........................................
ChangesNS   ..............................S............................N........................................

[           9301      9311      9321      9331      9341      9351      9361      9371      9381      9391      ]
[           |         |         |         |         |         |         |         |         |         |         ]
Dd636       TTTCTTTTGTTAGGAGTTGATTTTATTGCTTTAATGTTTTTGTTGGTTTATGTTGGGGCCATTGCTGTTTTATTTTTGTTTGTTATTATGTTGTTAAATT
Dd432       ....................................................................................................
Lp_KC875348 ....................................................................................................
Lp_KC875349 ....................................................................................................
Lp_FR821799 ....................................................................................................
ChangesNS   ....................................................................................................

[           9401      9411      9421      9431      9441      9451      9461      9471      9481      9491      ]
[           |         |         |         |         |         |         |         |         |         |         ]
Dd636       TAACGGATTATCCTCCTGCTTTTTTTAAAAGAGAGGCCGACATGACAAATTATATTCCGATTGGGTTCCTTATAGGTGTCTTTTTTTTTTCTGAGGCCTC
Dd432       .............C......................................................................................
Lp_KC875348 ....................................................................................................
Lp_KC875349 ....................................................................................................
Lp_FR821799 ....................................................................................................
ChangesNS   .............S......................................................................................
```

```
[           9501      9511      9521      9531      9541      9551      9561      9571      9581      9591       ]
[           |         |         |         |         |         |         |         |         |         |          ]
Dd636       TTCTAGTTGATTCGTTTTGGGACCTTTCCAAAAAGAGAACTGAGACTTGTCTTTTCCTTGACTTATTATTTCTTATCATAATATAGAGGCGCTGGGGCAA
Dd432       ...........................T........................................................................
Lp_KC875348 ...........................T.............................................C..........................
Lp_KC875349 ...........................T........................................................................
Lp_FR821799 ...........................T........................................................................
ChangesNS   ...........................S.............................................N..........................

[           9601      9611      9621      9631      9641      9651      9661      9671      9681      9691       ]
[           |         |         |         |         |         |         |         |         |         |          ]
Dd636       GTTTTATATGTTGTTTGTTGTTGTTTATTTATTTTGGCCAGCTTTATTCTTTTAGTTGCTATGCTCGGAGCTATTTTTTTAACTCAAGATATGATAAATA
Dd432       ..Y......................................................................................C..........
Lp_KC875348 ...............................................................A....................................
Lp_KC875349 ...............................................................A....................................
Lp_FR821799 ...............................................................A....................................
ChangesNS   ..S............................................................N.........................S..........

[           9701      9711      9721      9731      9741      9751      9761      9771      9781      9791       ]
[           |         |         |         |         |         |         |         |         |         |          ]
Dd636       AATATTTAATTCGTTGAGTTTTTTCTACAAACCACAAAGATATCGGTACTTTATATTTAGTTTTTGGGGTTGGGGCGGGTTTAATTGGAACGGCTTTTAG
Dd432       ....................................................................................................
Lp_KC875348 ....................................................................................................
Lp_KC875349 ....................................................................................................
Lp_FR821799 ....................................................................................................
ChangesNS   ....................................................................................................

[           9801      9811      9821      9831      9841      9851      9861      9871      9881      9891       ]
[           |         |         |         |         |         |         |         |         |         |          ]
Dd636       TATGCTTATACGACTGGAGCTTTCTGCGCCGGGGGCGATGCTGGGGGACGATCATCTTTATAATGTCATTGTAACAGCACATGCTTTTATTATGATTTTT
Dd432       ....................................................................................................
Lp_KC875348 ....................................................................................................
Lp_KC875349 ....................................................................................................
Lp_FR821799 ....................................................................................................
ChangesNS   ....................................................................................................

[           9901      9911      9921      9931      9941      9951      9961      9971      9981      9991       ]
[           |         |         |         |         |         |         |         |         |         |          ]
Dd636       TTTTTAGTTATGCCCGTTATGATTGGTGGGTTYGGTAATTGGTTGGTTCCACTATATATTGGAGCCCCTGATATGGCTTTCCCCCGATTAAACAATATTA
Dd432       ................................T...................................................................
Lp_KC875348 ................................T...................................................................
Lp_KC875349 ................................T...................................................................
Lp_FR821799 ................................T...................................................................
ChangesNS   ................................S...................................................................
```

```
[           10001     10011     10021     10031     10041     10051     10061     10071     10081     10091     ]
[           |         |         |         |         |         |         |         |         |         |         ]
Dd636       GTTTTTGGTTGTTGCCGCCGGCATTGTTTTTATTATTGGGTTCGGCTTTTGTAGAACAAGGAGTAGGAACAGGGTGAACGGTTTATCCCCCGTTGTCCAG
Dd432       ....................................................................................................
Lp_KC875348 .............................................T.................C....................................
Lp_KC875349 .............................................T.................C....................................
Lp_FR821799 .............................................T.................C....................................
ChangesNS   .............................................N.................N....................................

[           10101     10111     10121     10131     10141     10151     10161     10171     10181     10191     ]
[           |         |         |         |         |         |         |         |         |         |         ]
Dd636       TGTCCAAGCACACTCCGGGGGTTCTGTTGACATGGCGATTTTTAGTCTCCATTTGGCTGGGGCTTCTTCTATTTTAGGGGCAATAAACTTTATTACTACA
Dd432       ....................................................................................................
Lp_KC875348 ....................................................................................................
Lp_KC875349 ....................................................................................................
Lp_FR821799 .................................................................T..................................
ChangesNS   .................................................................N..................................

[           10201     10211     10221     10231     10241     10251     10261     10271     10281     10291     ]
[           |         |         |         |         |         |         |         |         |         |         ]
Dd636       ATTTTTAATATGCGGGCCCCGGGTATTACGTTTAATAAAATGCCTTTGTTTGTTTGGTCTATTTTAATCACTGCTTTTTTATTGCTTTTATCTTTACCTG
Dd432       ....................................................................................................
Lp_KC875348 ....................................................................................................
Lp_KC875349 ....................................................................................................
Lp_FR821799 ....................................................................................................
ChangesNS   ....................................................................................................

[           10301     10311     10321     10331     10341     10351     10361     10371     10381     10391     ]
[           |         |         |         |         |         |         |         |         |         |         ]
Dd636       TTTTAGCCGGTGCTATTACAATGCTTTTAACAGATCGTAATTTTAACACCACTTTTTTTGAGCCTTCAGGGGGGGGGGATCCGATTTTATTTCAGCATTT
Dd432       ....................................................................................................
Lp_KC875348 ....................................................................................................
Lp_KC875349 ....................................................................................................
Lp_FR821799 ....................................................................................................
ChangesNS   ....................................................................................................

[           10401     10411     10421     10431     10441     10451     10461     10471     10481     10491     ]
[           |         |         |         |         |         |         |         |         |         |         ]
Dd636       ATTTTGGTTTTTTGGACACCCAGAAGTTTATATTTTGATTTTACCGGGCTTTGGATGATCTCTCAAATTATTCCTACTTTTGTTGCAAAAAAACAAATT
Dd432       ....................................................................................................
Lp_KC875348 ....................................................................................................
Lp_KC875349 ....................................................................................................
Lp_FR821799 ....................................................................................................
ChangesNS   ....................................................................................................
```

```
[           10501     10511     10521     10531     10541     10551     10561     10571     10581     10591     ]
[           |         |         |         |         |         |         |         |         |         |         ]
Dd636       TTTGGATATTTAGGAATGGTTTATGCAATGCTTTCTATTGGGCTTCTTGGGTTTATTGTATGGGCACATCATATGTTTACTGTTGGTATGGATGTTGATA
Dd432       ....................................................................................................
Lp_KC875348 ....................................................................................................
Lp_KC875349 ....................................................................................................
Lp_FR821799 ....................................................................................................
ChangesNS   ....................................................................................................

[           10601     10611     10621     10631     10641     10651     10661     10671     10681     10691     ]
[           |         |         |         |         |         |         |         |         |         |         ]
Dd636       CAAGGGCATATTTTACAGCAGCAACGATGATAATTGCTGTTCCTACGGGGATTAAGGTTTTTAGTTGATTAGCTACTGTTTACGGGGGGGTTTTAAGATT
Dd432       ....................................................................................................
Lp_KC875348 ....................................................................................................
Lp_KC875349 ....................................................................................................
Lp_FR821799 ....................................................................................................
ChangesNS   ....................................................................................................

[           10701     10711     10721     10731     10741     10751     10761     10771     10781     10791     ]
[           |         |         |         |         |         |         |         |         |         |         ]
Dd636       AGACACTCCAATGCTTTGGGCTATGGGATTTGTTTTTTTATTTACATTAGGCGGGCTTACTGGGGTAATTTTAGCCAATAGTTCTCTTGATATTGTTCTT
Dd432       ....................................................................................................
Lp_KC875348 ....................................................................................................
Lp_KC875349 ....................................................................................................
Lp_FR821799 ....................................................................................................
ChangesNS   ....................................................................................................

[           10801     10811     10821     10831     10841     10851     10861     10871     10881     10891     ]
[           |         |         |         |         |         |         |         |         |         |         ]
Dd636       CATGATACATATTATGTTGTAGCACATTTTCATTATGTCCTTTCTATGGGGGCCGTGTTTGCTATCTTTGGGGGGTTTATTATTGAATCGGAAAAATTA
Dd432       ....................................................................................................
Lp_KC875348 ....................................................................................................
Lp_KC875349 ....................................................................................................
Lp_FR821799 ....................................................................................................
ChangesNS   ....................................................................................................

[           10901     10911     10921     10931     10941     10951     10961     10971     10981     10991     ]
[           |         |         |         |         |         |         |         |         |         |         ]
Dd636       GTGGTTATTGTTATAATGAGCTTTTTGGAAAAGTTCATTTTTGGTTGATGTTTATCGGAGTAAATTTAACTTTTTTCCCTCAACATTTTTTAGGTTTAAC
Dd432       ....................................................................................................
Lp_KC875348 ....................................................................................................
Lp_KC875349 ....................................................................................................
Lp_FR821799 ....................................................................................................
ChangesNS   ....................................................................................................
```

```
[            11001     11011     11021     11031     11041     11051     11061     11071     11081     11091      ]
[            |         |         |         |         |         |         |         |         |         |          ]
Dd636        GGGGTTCCCCAGACGATATTCGGATTTTGCAGATTCTTTTGCTGGTTGGAACCTAATTAGTTCTTTTGGTTCTGTTATTTCTATTTTAGGTGTTATATGA
Dd432        ....................................................................................................
Lp_KC875348  ....................................................................................................
Lp_KC875349  ....................................................................................................
Lp_FR821799  ....................................................................................................
ChangesNS    ....................................................................................................

[            11101     11111     11121     11131     11141     11151     11161     11171     11181     11191      ]
[            |         |         |         |         |         |         |         |         |         |          ]
Dd636        TTTTTATATCTTGTTTTTGACTTTTTTGTTTCAGAGGAAAGGTTTTTAGGTTGAAAAGGAGGAAGTTCTATAGAATGAAAACATTCTTCTCCTCCTGAGT
Dd432        ....................................................................................................
Lp_KC875348  ........................................A...........................................................
Lp_KC875349  ........................................A...........................................................
Lp_FR821799  ........................................A...........................................................
ChangesNS    ........................................N...........................................................

[            11201     11211     11221     11231     11241    ]
[            |         |         |         |         |    ]
Dd636        TTCACACTTATAACGWATTGCCTTTTGTGTTTAGAGTTCTAAGA
Dd432        ...............A............................
Lp_KC875348  ...............A............................
Lp_KC875349  ...............A............................
Lp_FR821799  ...............A......................T.....
ChangesNS    ...............S......................S.....
```
