# Supplementary material for: Merging scleractinian genera: the overwhelming genetic similarity between solitary Desmophyllum and colonial Lophelia
Source: BMC Evol Biol. 2016 May 18;16:108. doi: 10.1186/s12862-016-0654-8 (PMC4870751; doi:10.1186/s12862-016-0654-8)
Supplement: Additional file 3: — Plots showing the allele size frequency per loci of D. dianthus (blue) and L. pertusa (red) for the 30 new microsatellites markers developed and described in Addamo et al. [47]. (PDF 2595 kb) [file 12862_2016_654_MOESM3_ESM.pdf]

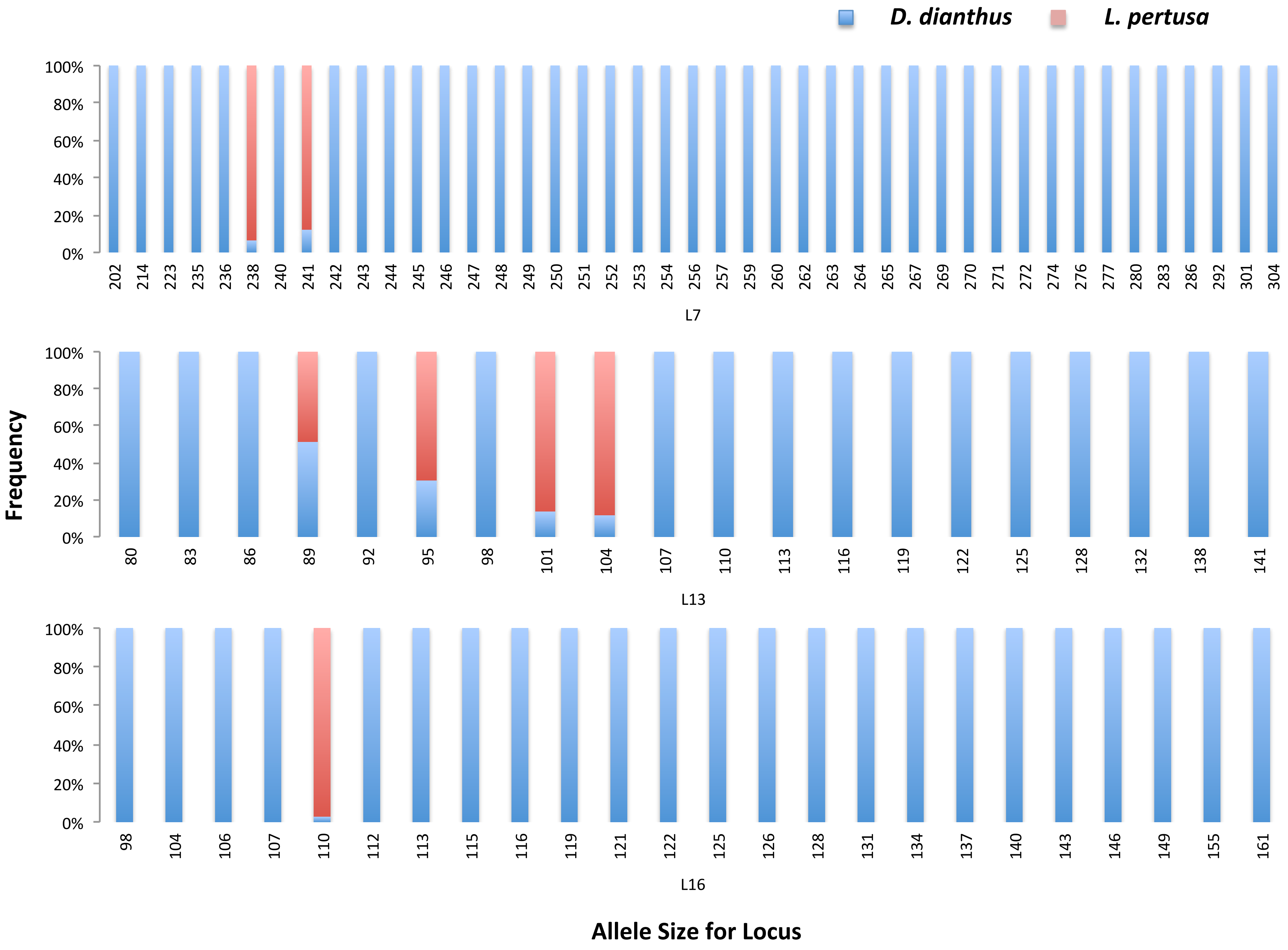
D. dianthus
L. pertusa
Frequency
100%
80%
60%
40%
20%
0%
202 214 223 235 236 238 240 241 242 243 244 245 246 247 248 249 250 251 252 253 254 256 257 259 260 262 263 264 265 267 269 270 271 272 274 276 277 280 283 286 292 301 304
L7
80 83 86 89 92 95 98 101 104 107 110 113 116 119 122 125 128 132 138 141
L13
98 104 106 107 110 112 113 115 116 119 121 122 125 126 128 131 134 137 140 143 146 149 155 161
L16
Allele Size for Locus

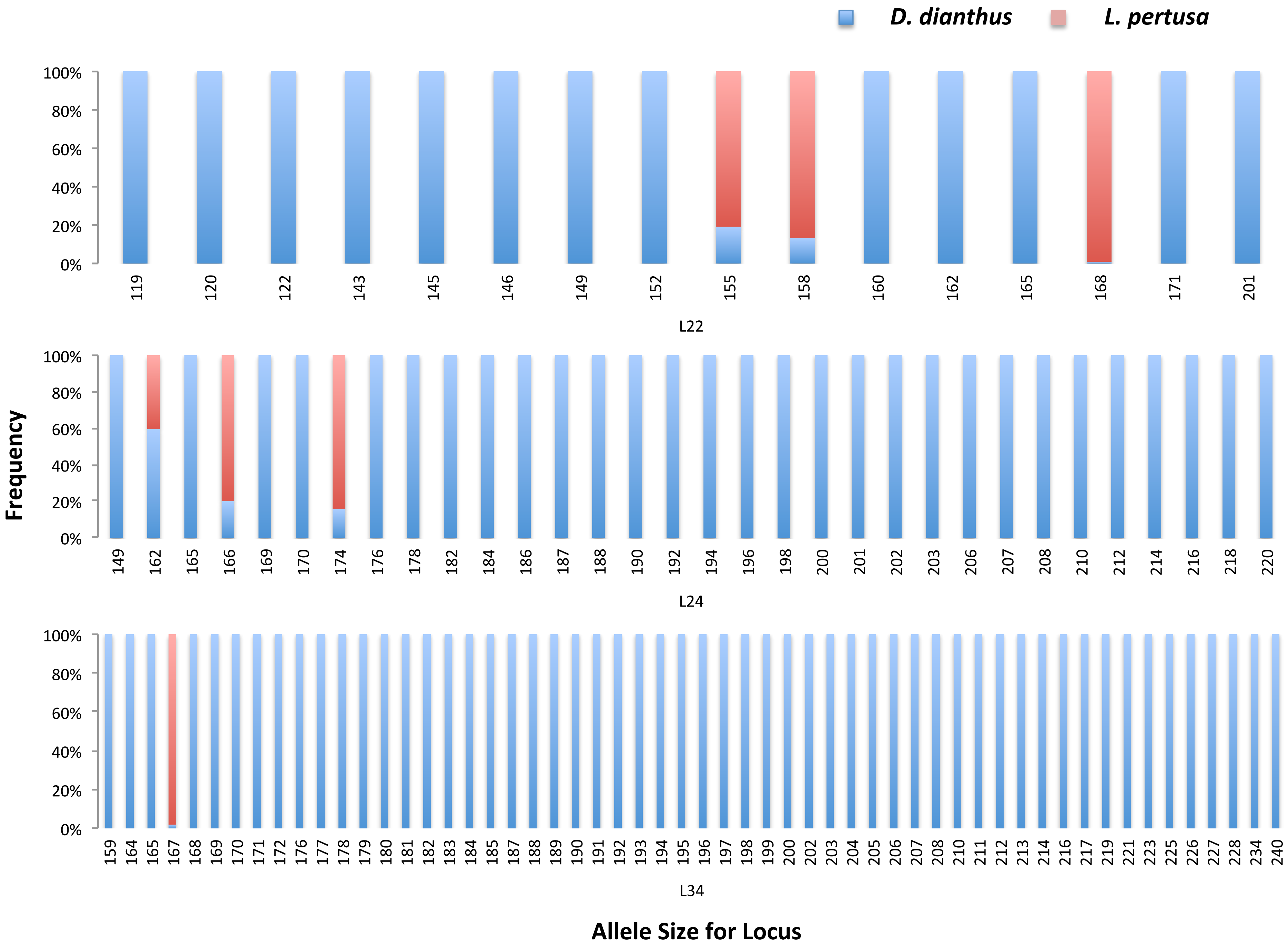

D. dianthus
L. pertusa
Frequency
100%
80%
60%
40%
20%
0%
119 120 122 143 145 146 149 152 155 158 160 162 165 168 171 201
L22
149 162 165 166 169 170 174 176 178 182 184 186 187 188 190 192 194 196 198 200 201 202 203 206 207 208 210 212 214 216 218 220
L24
159 164 165 167 168 169 170 171 172 176 177 178 179 180 181 182 183 184 185 187 188 189 190 191 192 193 194 195 196 197 198 199 200 202 203 204 205 206 207 208 210 211 212 213 214 216 217 219 221 223 225 226 227 228 234 240
L34
Allele Size for Locus

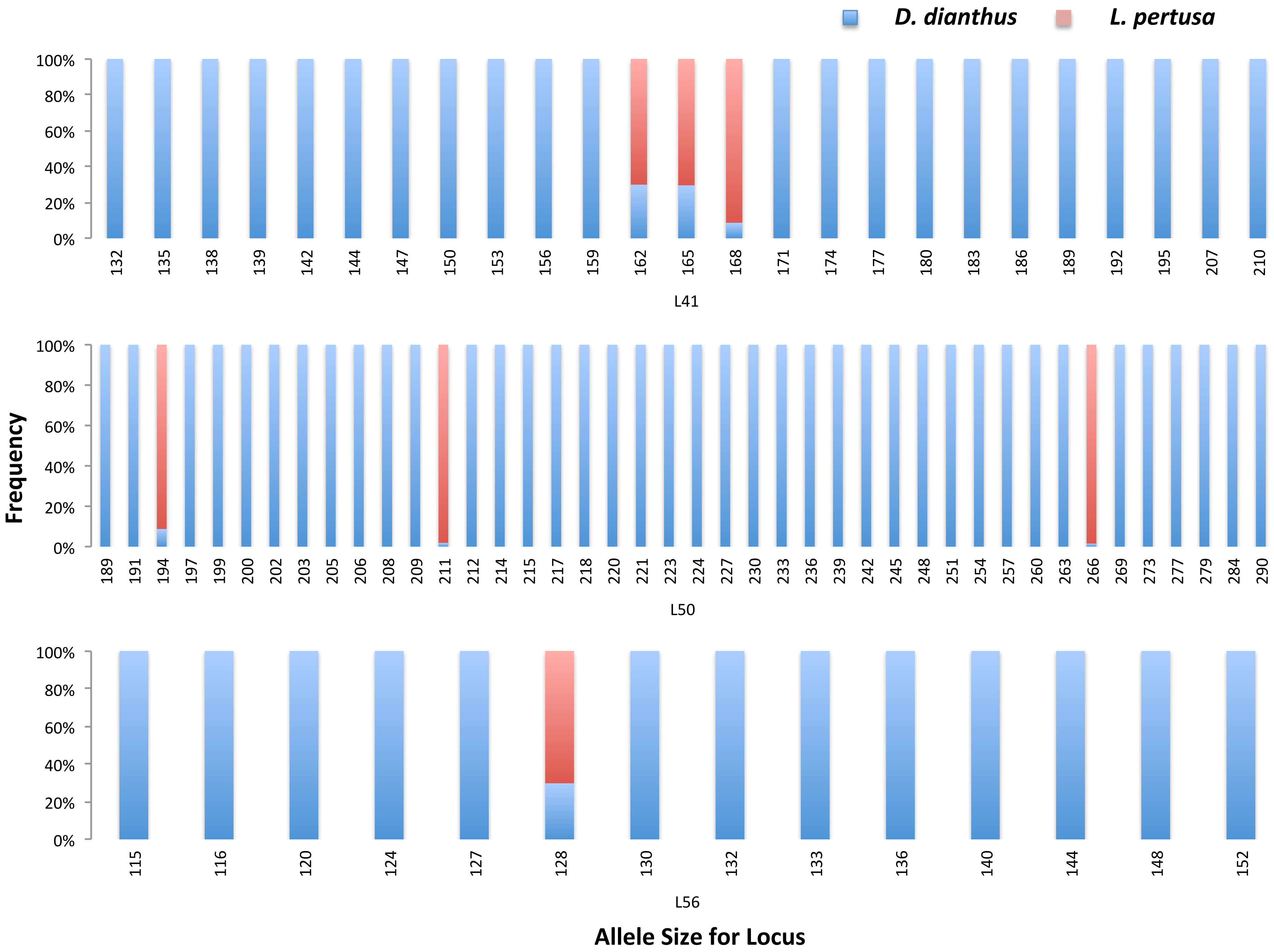

D. dianthus
L. pertusa
Frequency
100%
80%
60%
40%
20%
0%
132 135 138 139 142 144 147 150 153 156 159 162 165 168 171 174 177 180 183 186 189 192 195 207 210
L41
189 191 194 197 199 200 202 203 205 206 208 209 211 212 214 215 217 218 220 221 223 224 227 230 233 236 239 242 245 248 251 254 257 260 263 266 269 273 277 279 284 290
L50
115 116 120 124 127 128 130 132 133 136 140 144 148 152
L56
Allele Size for Locus

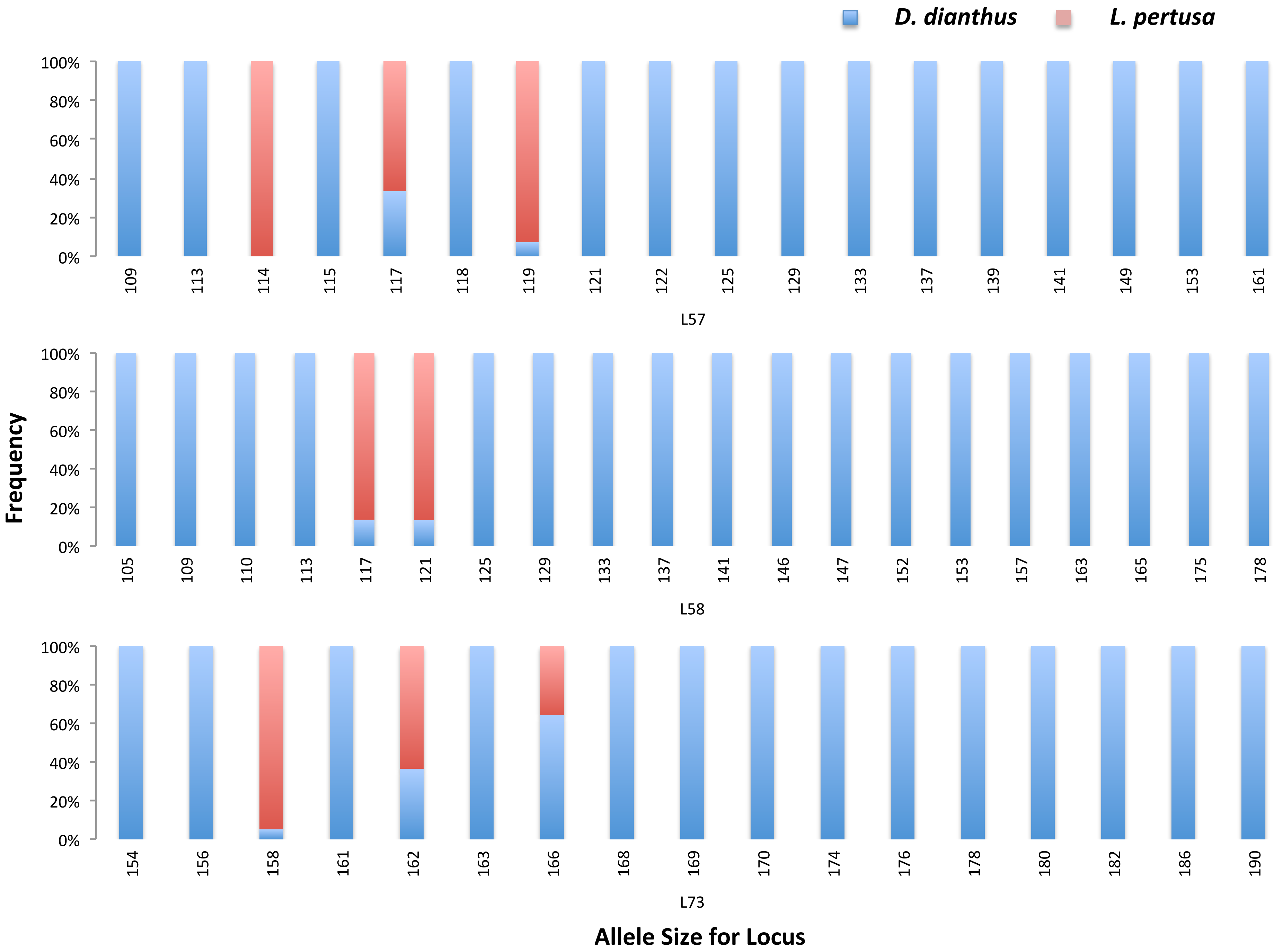
D. dianthus
L. pertusa
Frequency
100%
80%
60%
40%
20%
0%
109 113 114 115 117 118 119 121 122 125 129 133 137 139 141 149 153 161
L57
105 109 110 113 117 121 125 129 133 137 141 146 147 152 153 157 163 165 175 178
L58
154 156 158 161 162 163 166 168 169 170 174 176 178 180 182 186 190
L73
Allele Size for Locus

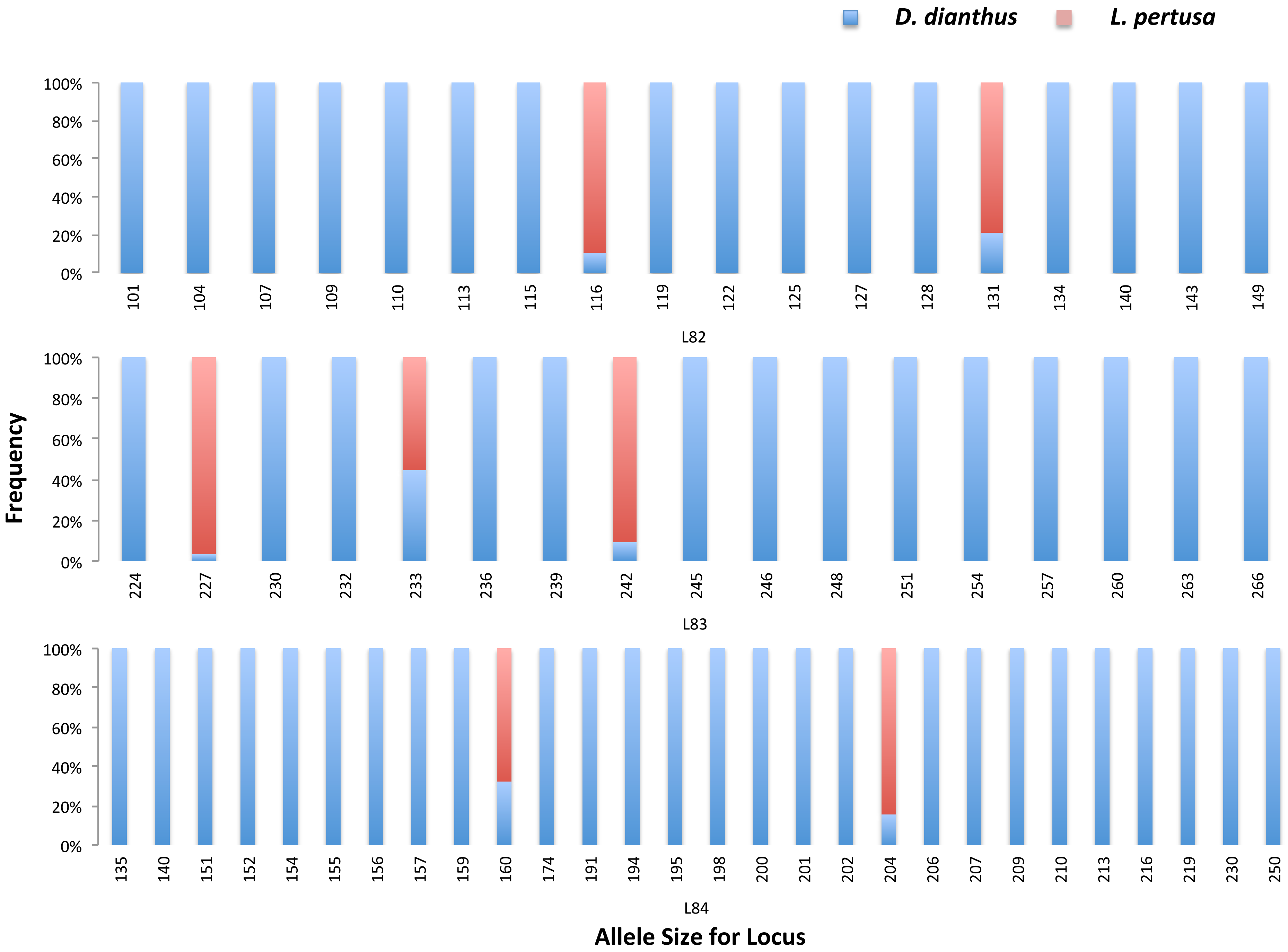
D. dianthus
L. pertusa
Frequency
100%
80%
60%
40%
20%
0%
101 104 107 109 110 113 115 116 119 122 125 127 128 131 134 140 143 149
L82
224 227 230 232 233 236 239 242 245 246 248 251 254 257 260 263 266
L83
135 140 151 152 154 155 156 157 159 160 174 191 194 195 198 200 201 202 204 206 207 209 210 213 216 219 230 250
L84
Allele Size for Locus

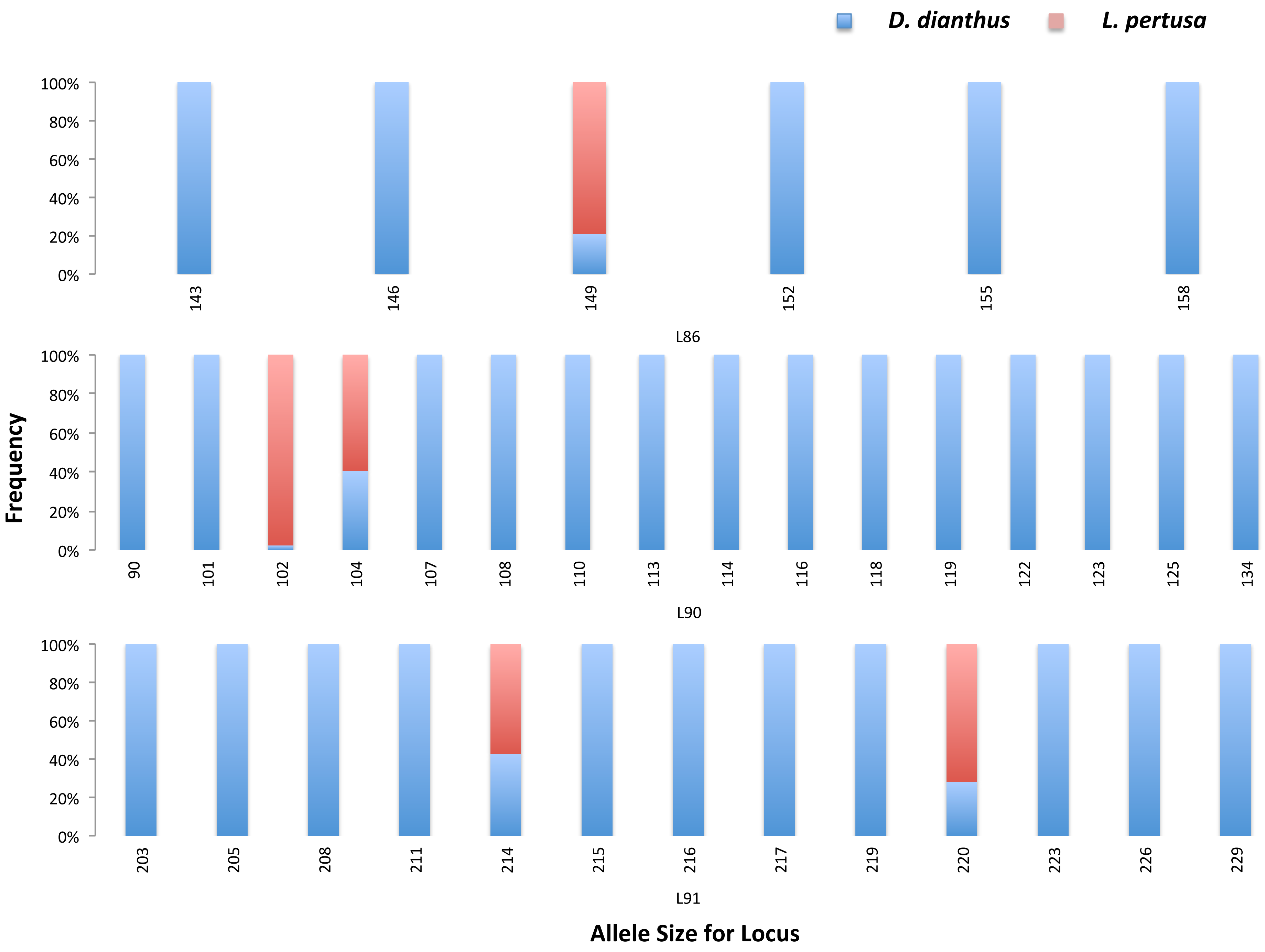
D. dianthus
L. pertusa
Frequency
100%
80%
60%
40%
20%
0%
143
146
149
152
155
158
L86
90
101
102
104
107
108
110
113
114
116
118
119
122
123
125
134
L90
203
205
208
211
214
215
216
217
219
220
223
226
229
L91
Allele Size for Locus

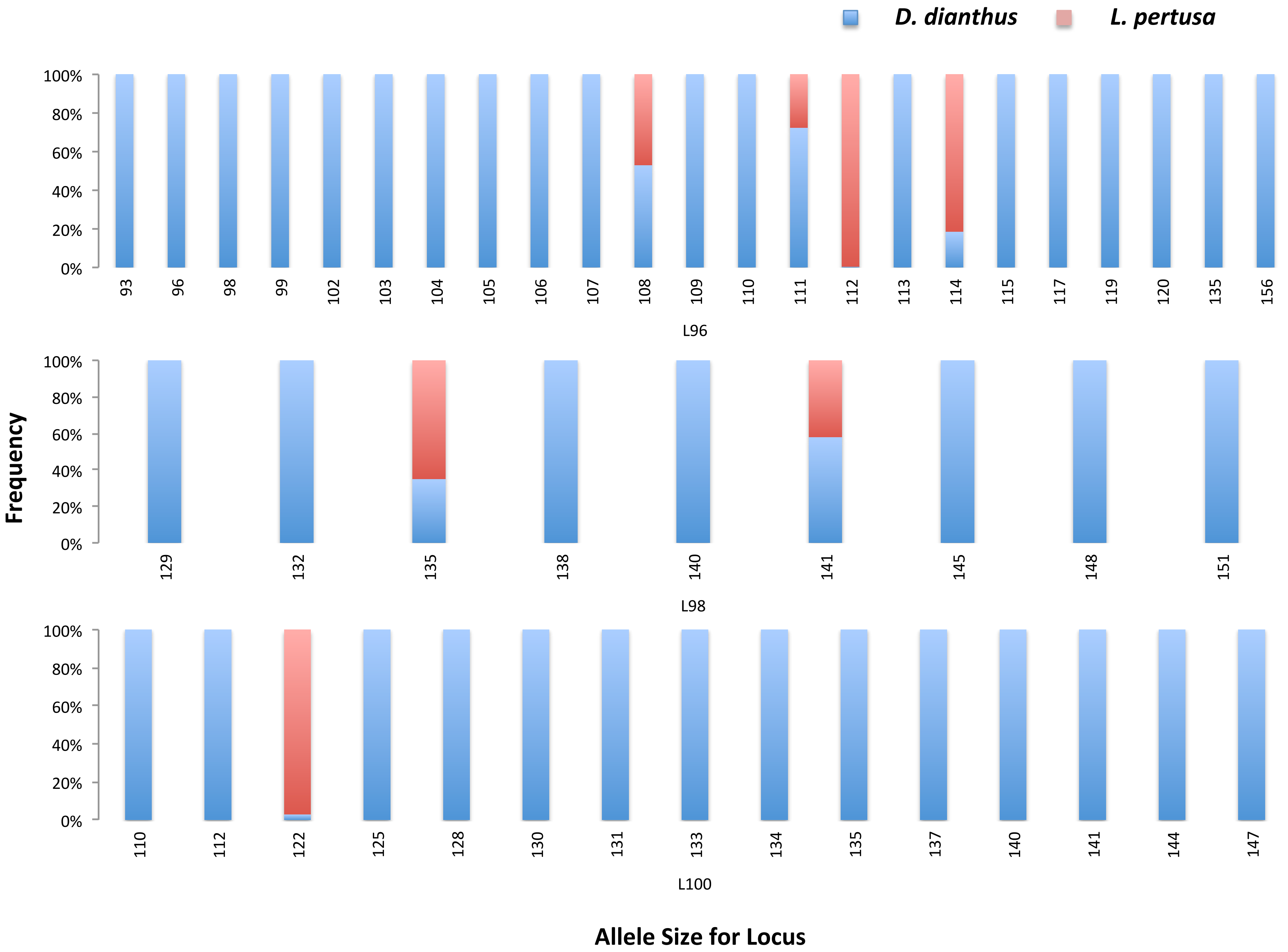

D. dianthus
L. pertusa
100%
80%
60%
40%
20%
0%
93 96 98 99 102 103 104 105 106 107 108 109 110 111 112 113 114 115 117 119 120 135 156
L96
129 132 135 138 140 141 145 148 151
L98
110 112 122 125 128 130 131 133 134 135 137 140 141 144 147
L100
Frequency
Allele Size for Locus

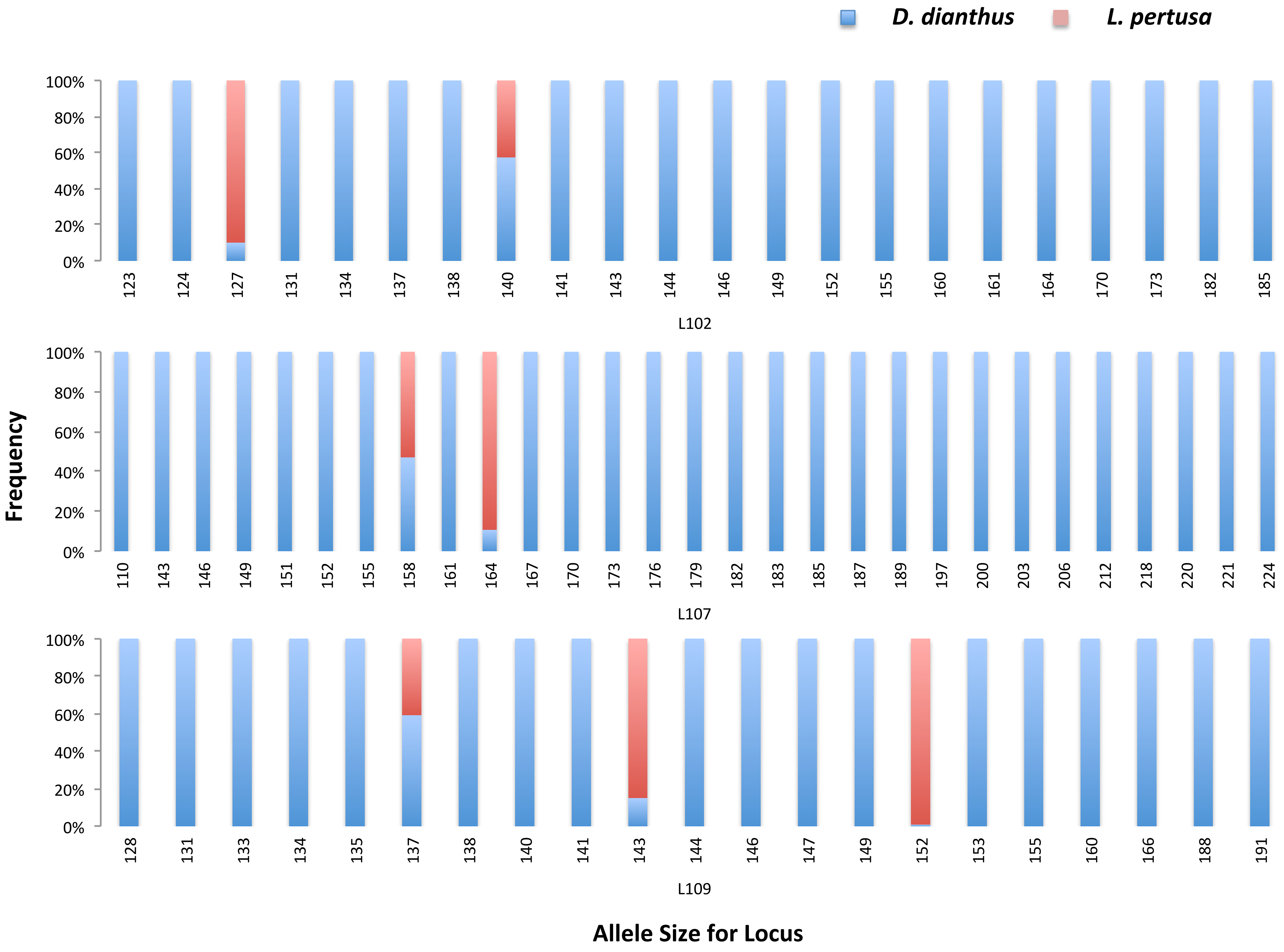
D. dianthus
L. pertusa
Frequency
100%
80%
60%
40%
20%
0%
123 124 127 131 134 137 138 140 141 143 144 146 149 152 155 160 161 164 170 173 182 185
L102
110 143 146 149 151 152 155 158 161 164 167 170 173 176 179 182 183 185 187 189 197 200 203 206 212 218 220 221 224
L107
128 131 133 134 135 137 138 140 141 143 144 146 147 149 152 153 155 160 166 188 191
L109
Allele Size for Locus

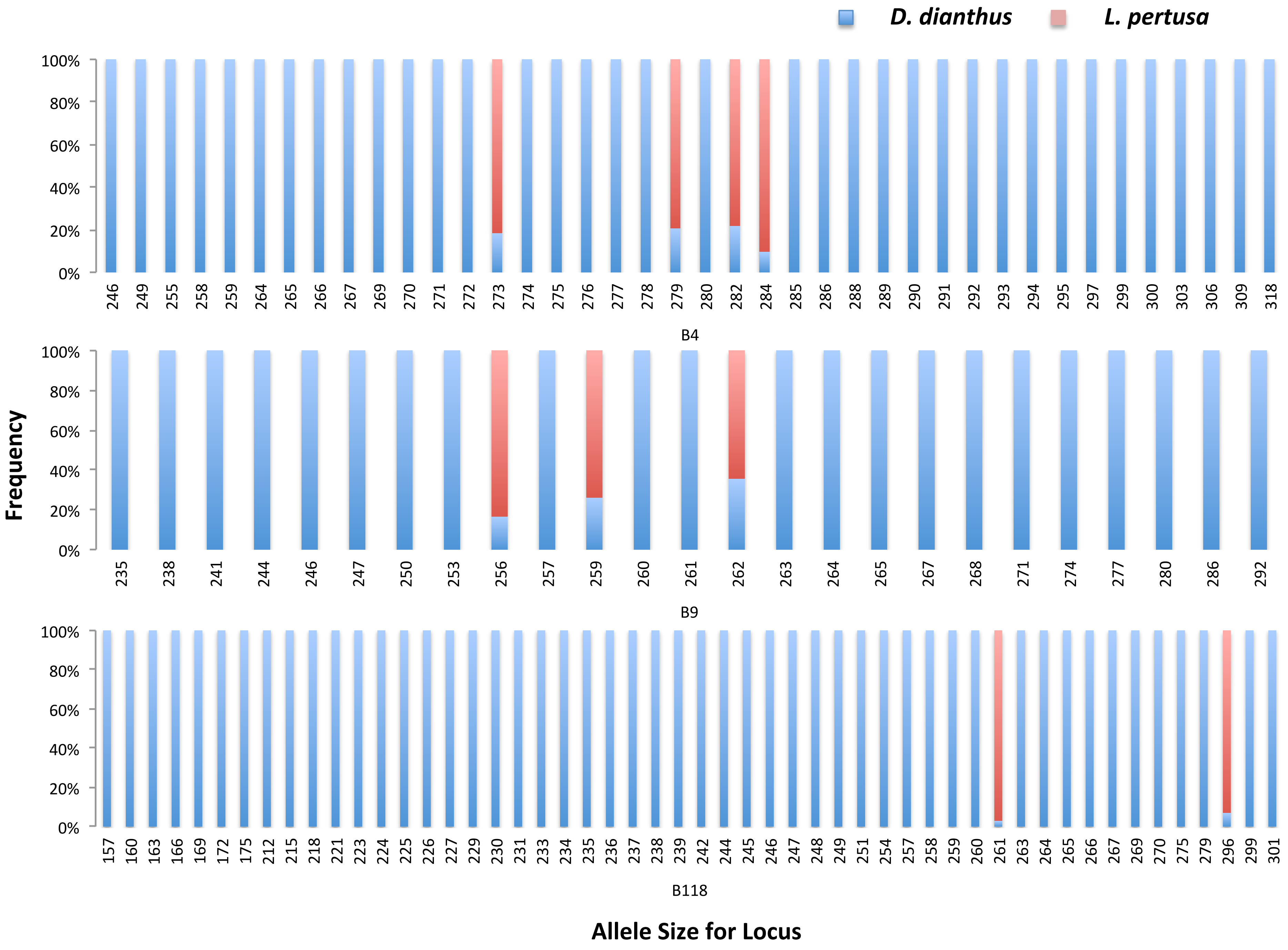
D. dianthus
L. pertusa
Frequency
100%
80%
60%
40%
20%
0%
246 249 255 258 259 264 265 266 267 269 270 271 272 273 274 275 276 277 278 279 280 282 284 285 286 288 289 290 291 292 293 294 295 297 299 300 303 306 309 318
B4
235 238 241 244 246 247 250 253 256 257 259 260 261 262 263 264 265 267 268 271 274 277 280 286 292
B9
157 160 163 166 169 172 175 212 215 218 221 223 224 225 226 227 229 230 231 233 234 235 236 237 238 239 242 244 245 246 247 248 249 251 254 257 258 259 260 261 263 264 265 266 267 269 270 275 279 296 299 301
B118
Allele Size for Locus

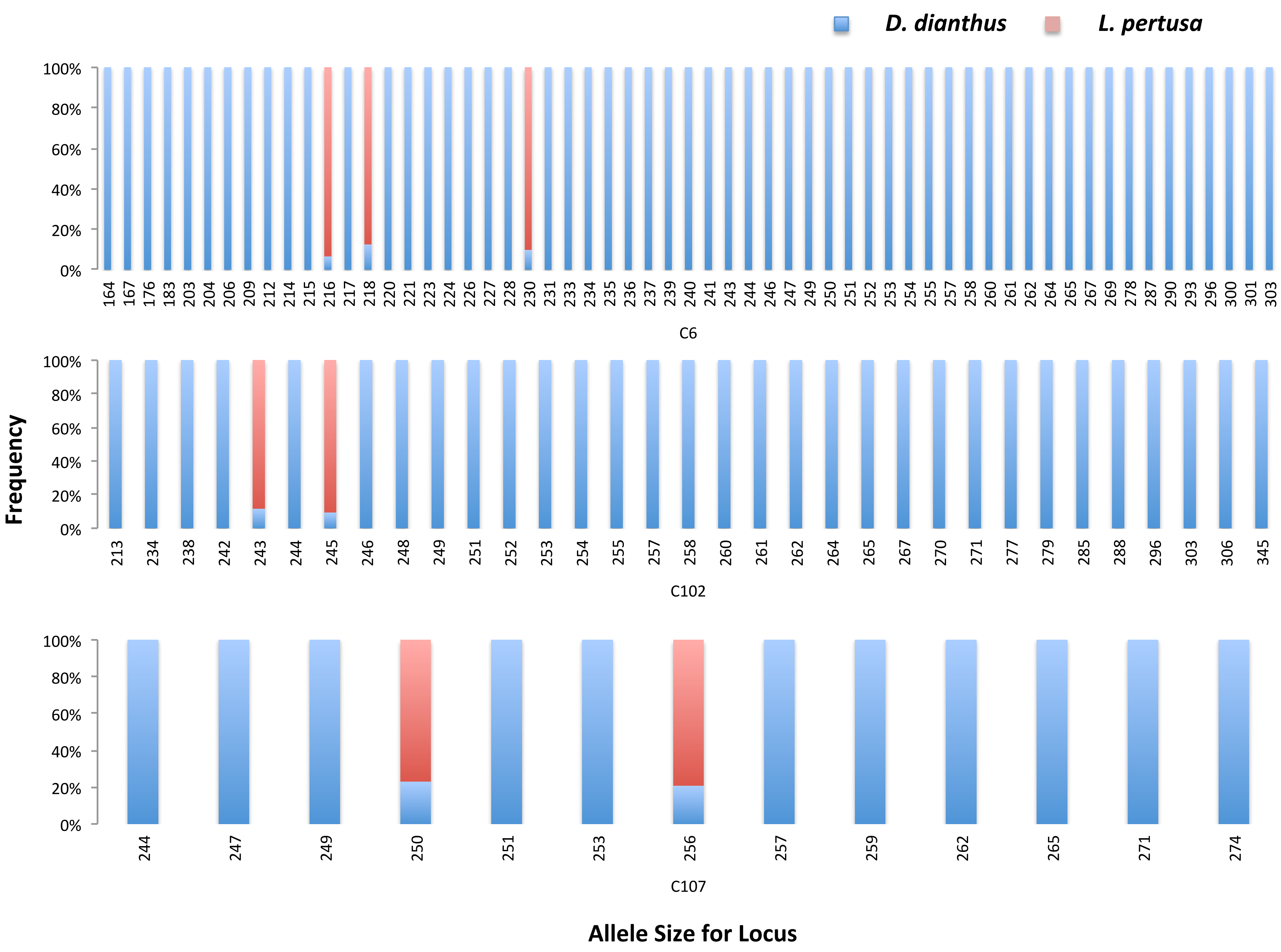
D. dianthus
L. pertusa
Frequency
100%
80%
60%
40%
20%
0%
164 167 176 183 203 204 206 209 212 214 215 216 217 218 220 221 223 224 226 227 228 230 231 233 234 235 236 237 239 240 241 243 244 246 247 249 250 251 252 253 254 255 257 258 260 261 262 264 265 267 269 278 287 290 293 296 300 301 303
C6
213 234 238 242 243 244 245 246 248 249 251 252 253 254 255 257 258 260 261 262 264 265 267 270 271 277 279 285 288 296 303 306 345
C102
244 247 249 250 251 253 256 257 259 262 265 271 274
C107
Allele Size for Locus
