# Supplementary figures and images for: Merging scleractinian genera: the overwhelming genetic similarity between solitary Desmophyllum and colonial Lophelia
Source: BMC Evol Biol. 2016 May 18;16:108. doi: 10.1186/s12862-016-0654-8 (PMC4870751; doi:10.1186/s12862-016-0654-8)

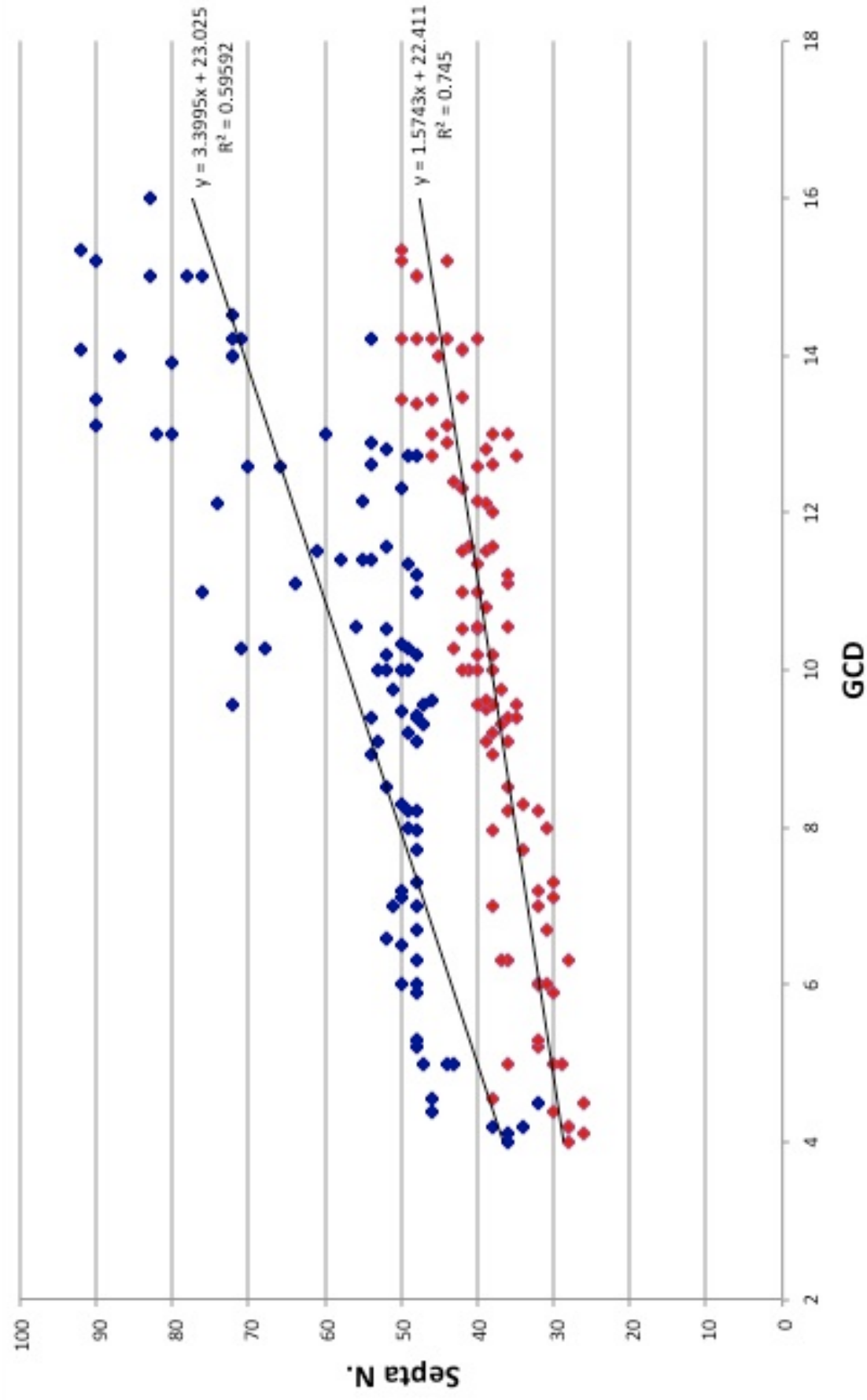

Supplement: Additional file 4: — Plot showing the relationship between the Greater Calicular Diameter (GCD in mm) and number of septa (S) in juvenile coralla of D. dianthus (blue diamonds) and L. pertusa (red diamonds). At equal GCD, the number of septa is normally higher in D. dianthus than in L. pertusa; there is also larger variability in septal number at a given GCD in D. dianthus than L. pertusa. (PDF 61 kb) [file 12862_2016_654_MOESM4_ESM.pdf]

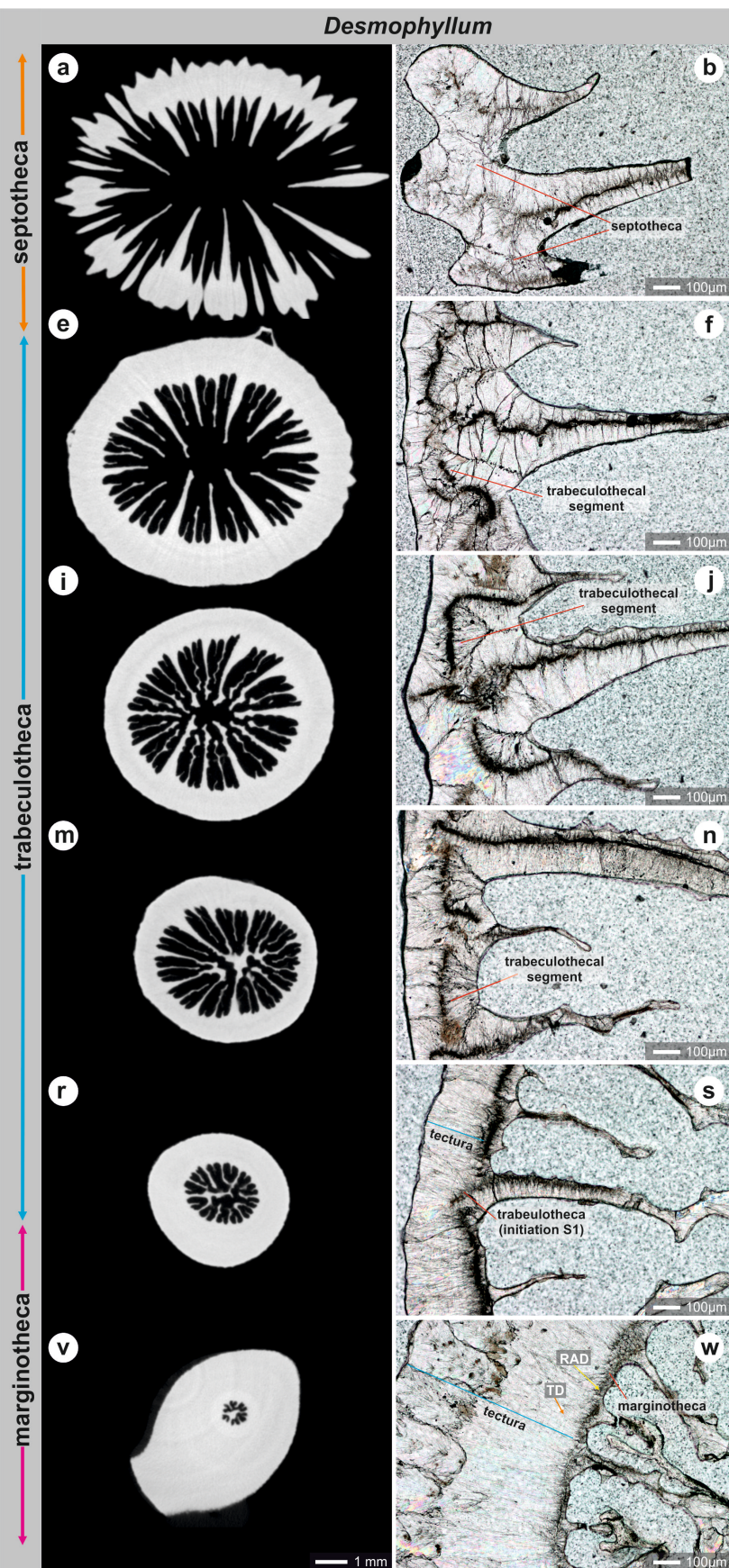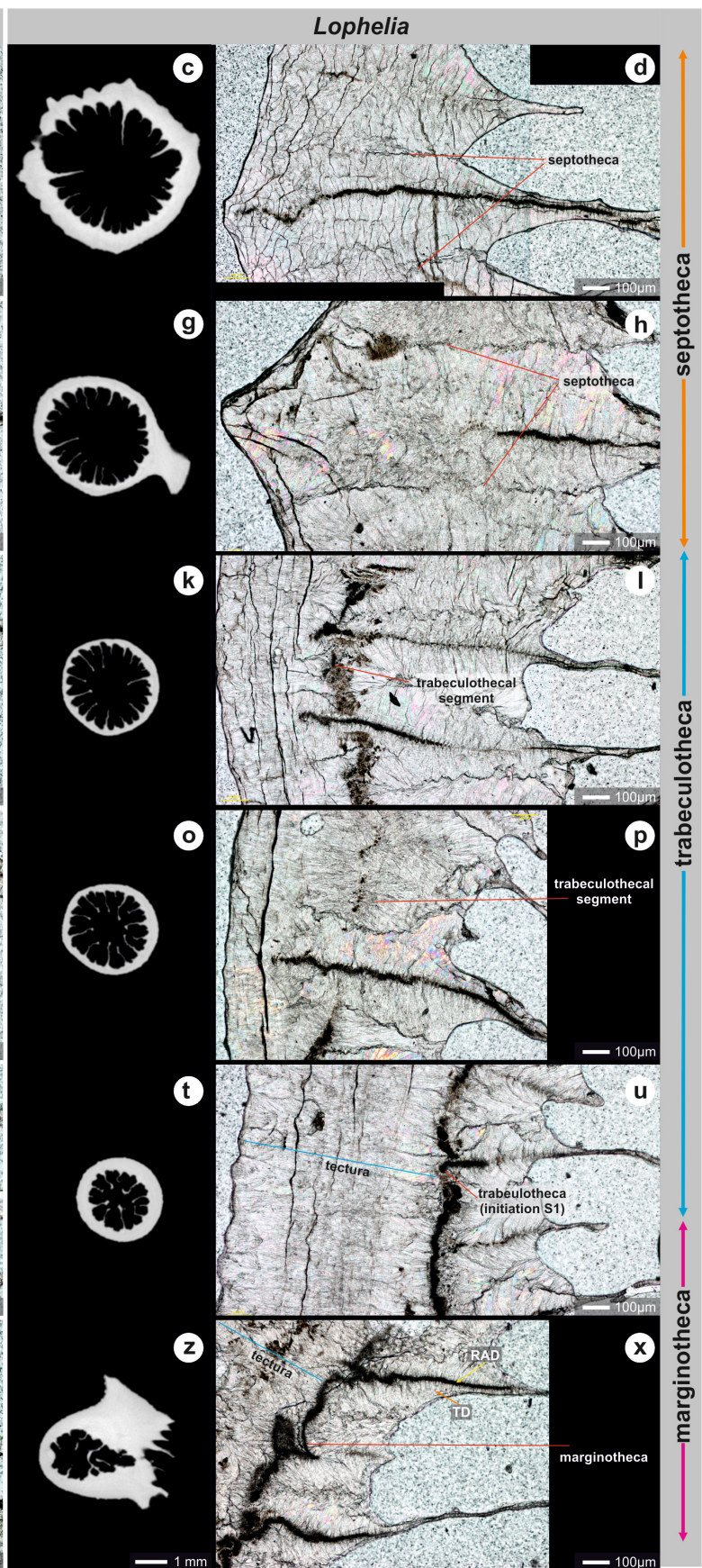

Supplement: Additional file 5: — Ontogenetic and microstructural skeletal features of Desmophyllum dianthus (Dd ROC 180, a, e, i, m, r, v—virtual mCT sections; b, f, j, n, s, w—thin sections, transmitted light microscope) and Lophelia pertusa (MEDCOR 09, c, g, k, o, t, z—virtual mCT sections; d, h, l, p, u, x—thin sections, transmitted light microscope). In both taxa, spatial relationships between the septa and wall transform similarly during ontogeny, described as a thecal sequence from marginotheca (red arrows) to trabeculotheca (blue arrows) to septotheca (orange arrows in vertical columns). Virtual mCT sections (left column for each taxon) correspond to the phases of growth depicted in transverse thin sections (right column for each taxon). Septotheca typically develops slightly later in the ontogeny of Desmophyllum (see length of orange arrows). Microstructural organization of coralla of both taxa is very similar and simple: septa and wall consist of densely packed Rapid Accretion Deposits, RAD (traditional "centers of calcification") and Thickening Deposits, TD (traditional fibers), which radiate outward (in transverse sections) from RADs. (PDF 12352 kb) [file 12862_2016_654_MOESM5_ESM.pdf]
